# Effectiveness of Acceptance and Commitment Therapy (ACT) on disease acceptance for breast cancer patients: Study protocol of a randomized controlled trial

**DOI:** 10.1371/journal.pone.0312669

**Published:** 2024-11-11

**Authors:** Wenjun Song, Nurul Izzah Shari, Jinggui Song, Ruiling Zhang, Nor Shuhada Mansor, Mohammad Farris Iman Leong Bin Abdullah, Zhaohui Zhang

**Affiliations:** 1 The First Affiliated Hospital of Xinxiang Medical University, Xinxiang, Henan, People’s Republic of China; 2 The Second Affiliated Hospital of Xinxiang Medical University, Xinxiang, Henan, People’s Republic of China; 3 Department of Community Health, Advanced Medical and Dental Institute, Universiti Sains Malaysia, Kepala Batas, Pulau Pinang, Malaysia; 4 Faculty of Social Sciences and Humanities, School of Human Resource Development and Psychology, Universiti Teknologi Malaysia, Skudai, Johor, Malaysia; 5 Department of Psychiatry, Faculty of Medicine, Universiti Sultan Zainal Abidin, Kuala Terengganu, Terengganu, Malaysia; The Chinese University of Hong Kong, HONG KONG

## Abstract

**Background:**

Breast cancer patients face significant psychological challenges, including difficulties in accepting the diagnosis, treatment, and long-term impact of the disease. Acceptance and Commitment Therapy (ACT) has shown promise in enhancing acceptance and psychological flexibility in various populations. This study aims to investigate the effectiveness of ACT in promoting disease acceptance among breast cancer patients through a randomized controlled trial.

**Methods:**

This study will recruit 90 breast cancer patients and randomly allocate them to an ACT intervention or control group. The ACT intervention, focusing on acceptance, mindfulness, value clarification, and committed action, will be delivered over 4 weeks. Meanwhile, the control group will receive standard care with non-therapeutic intervention. The study’s primary outcome is disease acceptance, while secondary outcomes include depression, anxiety, social support, quality of life (QoL), and psychological inflexibility. Data will be collected at three points: baseline, post-intervention, and three-month follow-up. Statistical analysis will compare outcomes between groups to evaluate the effectiveness and mechanism of this intervention using covariance and mediation analysis.

**Discussion:**

This study evaluates the effectiveness of ACT in promoting disease acceptance among breast cancer patients. It hypothesizes that the ACT group will show higher disease acceptance and improvements in social support, QoL, and psychological flexibility compared to the control group. The findings will contribute to research on psychological interventions and demonstrate ACT’s effectiveness in enhancing disease acceptance.

**Trial registration:**

The research project is registered in the ClinicalTrials (NCT05327153).

## Introduction

Breast cancer is a widespread and impactful health issue that significantly impacts the lives of numerous women across the globe. By 2020, there will be 2,179,457 new breast cancer cases in women globally, making up 24% of all new cancer cases in women, predicts the World Health Organization (WHO). Contrarily, breast cancer has a good prognosis and a protracted survival period. In some developed regions, the five-year standardized relative survival rate for breast cancer exceeds 80% [1]. Breast cancer has a high incidence, but its survival rate is generally more significant than most other cancers, which means that long-term survival rates are improving. However, most patients’ QoL is poor [2]. In addition to the physical hurdles, breast cancer frequently leads to significant psychological distress and emotional upheaval among patients. Malignant tumors are often linked to death in the minds of patients. Patients are under great psychological stress while dealing with the physical discomfort caused by the condition. Anxiety and despair are common negative emotions [3]. The psychological state of cancer patients is influenced by the diagnosis, complications, and treatment side effects [4]. Breast cancer patients had a greater rate of mental disorders after diagnosis than the general population, with 42% meeting rigorous diagnostic criteria for mental disorders within four weeks of diagnosis, the highest rate of all cancers [5]. Additionally, breast cancer patients are more likely to experience subclinical psychological problems and symptomatic psychological discomfort, with frequencies ranging from 30% to 75% [6]. The impact of these psychological problems on patients’ QoL is profound. Psychological distress can exacerbate physical symptoms, interfere with treatment adherence, and negatively influence overall well-being and recovery [7]. Anxiety and depression can lead to decreased motivation, social withdrawal, and impaired cognitive functioning in daily activities [8]. Furthermore, negative emotions can strain personal relationships and support systems, making it more challenging for patients to cope with their diagnosis and treatment [9]. Therefore, finding ways to enhance the psychological rehabilitation of breast cancer patients is crucial in oncology. Effective psychological support and interventions are essential to improve the overall QoL and aid in the holistic recovery of breast cancer patients.

Disease acceptance, which refers to the capacity to recognise and adjust to breast cancer’s diagnosis, treatment, and long-term consequences, significantly influences patients’ overall well-being and adaptation [10]. However, many individuals diagnosed with breast cancer struggle to accept their condition. These difficulties include dealing with the fear of recurrence, coping with body image changes due to surgery and treatments, managing the side effects of chemotherapy and radiation, and financial stress [11]. These challenges contribute to increased emotional distress, reduced QoL, and difficulties in effectively managing their treatment and survivorship. According to a recent study, 20% of breast cancer patients following mastectomy still struggle to accept their diagnosis [12]. Empirical research on acceptance intervention found that those who underwent it and were exposed to unpleasant interoceptive stimuli exhibited less behavioral avoidance, intense dread, cognitive panic symptoms, and catastrophic thoughts [13]. Good acceptance can effectively reduce individual anxiety levels [14]. Furthermore, due to the shift in the modern medical model, breast cancer treatment now focuses on extending patients’ survival times, lowering mortality, and improving their QoL [15]. Meanwhile, social support can encourage breast cancer patients to utilize positive coping strategies, increase their cognitive understanding of the disease, boost their self-esteem, and improve their QoL [16]. The most important aspect of the adaptation process is accepting the disease. Better disease acceptance can lead to less stress and higher self-esteem, making adjusting to new health statuses easier. As a result, this study aims to see if boosting illness acceptance improves breast cancer patients’ psychological well-being.

In response to the psychological effects of breast cancer, researchers and practitioners have investigated a range of therapeutic approaches. Studies have shown that psychotherapy may reduce cancer patients’ psychological suffering, ease their anxiety and sadness, and enhance their QoL [17]. The development of psycho-oncology has led to increased use of psychological interventions in breast cancer patients, recognizing the significant impact of mental health on overall well-being and treatment outcomes [18–20]. Several psychological interventions have been employed to support breast cancer patients, including cognitive-behavioral therapy (CBT), mindfulness-based stress reduction (MBSR), supportive-expressive therapy, and psychodynamic therapy. These interventions aim to address various aspects of psychological distress, such as anxiety, depression, fear of recurrence, and body image concerns, thereby improving patients’ QoL and ability to cope with their diagnosis and treatment [21]. Among these approaches, ACT stands out.

ACT is a contemporary form of cognitive-behavioral therapy that emphasizes the importance of psychological flexibility. This flexibility is the ability to fully engage with the present moment and persist or change behavior in a way that aligns with personal values, even in the presence of challenging thoughts and feelings. Grounded in Relational Frame Theory (RFT) [22], ACT incorporates mindfulness and acceptance strategies alongside commitment and behavior change techniques to help individuals live a more meaningful and value-driven life. The six core processes of ACT are acceptance, cognitive defusion, being present, self as context, values, and committed action. Together, these processes aim to reduce the influence of unhelpful thoughts and feelings, enabling individuals to pursue their goals and values more effectively [23].

ACT has demonstrated feasibility and promise across various contexts for breast cancer patients. It has shown potential in improving fatigue and sleep-related outcomes in breast cancer patients [24]. ACT is also effective in managing fear of cancer recurrence and associated significant improvements in avoidant coping, anxiety, depression, and QoL [25]. Additionally, ACT has proven to be an effective intervention for reducing depression and enhancing psychological flexibility among breast cancer patients [26]. Even a single-session ACT intervention has yielded small but positive improvements in postsurgical pain and anxiety, reinforcing its utility in this population [27]. ACT may enhance disease acceptance by encouraging patients to accept their negative emotions, thoughts, and physical sensations related to their cancer diagnosis and treatment, thereby reducing the impact of these negative thoughts and feelings on their daily lives. By fostering acceptance and mindfulness, ACT helps patients reframe their perception of the stressors associated with breast cancer, viewing them as manageable aspects of their lives rather than overwhelming threats. Furthermore, ACT emphasizes identifying personal values and committing to actions consistent with these values, even in the face of discomfort. This value-driven focus can reduce the perceived burden of the disease and improve patients’ overall QoL, making it easier for them to accept their condition and its long-term consequences. By promoting psychological inflexibility, ACT enables patients to adapt more effectively to their illness, facilitating better disease acceptance and QoL.

In one study, ACT is used to help breast cancer patients feel better about their lives and mental health. Jing Han et al. (2009) showed that ACT intervention effectively enhanced psychological inflexibility, disease cognition (including acceptance), and QoL [28]. However, no randomized controlled trial was carried out to verify the outcomes of this early investigation. This study aims to close that knowledge gap by conducting an RCT to evaluate the effectiveness of ACT in improving psychological inflexibility, disease acceptance, and QoL among breast cancer patients. Specifically, it seeks to provide fresh perspectives on how enhancing acceptance of the diagnosis through ACT can directly impact the QoL in breast cancer patients. Although there is strong evidence linking breast cancer patients’ psychological inflexibility, depression, anxiety, and QoL, the process is still not fully understood [29]. This study aims to investigate how ACT enhances the QoL in breast cancer patients by examining the potential mediating factors of psychological inflexibility and disease acceptance. The findings of the mediation analysis will help establish evidence for the mechanism of action for ACT’s efficacy.

Moreover, social support can encourage patients to use positive coping strategies and reduce the use of negative ones. A study involving 90 breast cancer patients administered supportive intervention revealed significant improvements in body image, physical and mental health, and various functions [30]. Therefore, in our study, social support will be a moderator variable to illustrate the change in illness cognition, psychological inflexibility, depression, anxiety, and QoL according to ACT intervention, thus further elucidating the mechanism of change. Overall, the results of the mediation and moderation analysis will contribute to the advancement of knowledge, the development of novel theories, and the enhancement of ACT treatment efficacy to patient requirements.

Overall, our study aims to assess the ACT intervention thoroughly, bridge the existing gap in the literature, and offer evidence-based suggestions for healthcare professionals and clinicians who support individuals with breast cancer. Ultimately, our findings have the potential to guide the development of tailored interventions that enhance disease acceptance, improve psychological well-being, and contribute to the long-term adjustment and overall QoL for breast cancer patients.

## Objective

### Primary objective

1. To compare the efficacy of ACT in enhancing disease acceptance compared with the waitlist control group across three-time points (T0 = baseline assessment, T1 = 4 weeks, immediately after completion of the intervention, T2 = 6 months after commencement of the intervention, and T3 = 12 months after commencement of the intervention).

### Secondary objectives

1. To compare the efficacy of ACT in reducing psychological inflexibility with the waitlist control group across three-time points (T0 = baseline assessment, T1 = 4 weeks, immediately after completion of the intervention, T2 = 6 months after commencement of the intervention, and T3 = 12 months after commencement of the intervention).
2. To compare the efficacy of ACT in reducing depression and anxiety compared with the waitlist control group across three-time points (T0 = baseline assessment, T1 = 4 weeks immediately after completion of the intervention, T2 = 6 months after commencement of the intervention, and T3 = 12 months after commencement of the intervention).
3. To compare the efficacy of ACT in enhancing QoL compared with the waitlist control group across three-time points (T0 = baseline assessment, T1 = 4 weeks, immediately after completion of the intervention, T2 = 6 months after commencement of the intervention, and T3 = 12 months after commencement of the intervention).
4. To compare the efficacy of ACT in enhancing social support compared with the waitlist control group across three-time points (T0 = baseline assessment, T1 = 4 weeks, immediately after completion of the intervention, T2 = 6 months after commencement of the intervention, and T3 = 12 months after commencement of the intervention).
5. To evaluate the mediating role of psychological inflexibility and disease acceptance in the relationship between ACT and QoL in breast cancer patients following ACT intervention.
6. To assess the moderating role of social support in the relationship between ACT and Psychological inflexibility in breast cancer patients.

## Methods

### Ethic

Ethical approval for this study was obtained from the Jawatankuasa Etika Penyelidikan Manusia, Universiti Sains Malaysia (JEPeM-USM), with approval granted on 19th December 2022 (JEPeM Code: USM/JEPeM/22080569). The approval was subsequently extended from 19th December 2023 to 18th December 2024. All procedures performed in studies involving human participants were in accordance with the ethical standards of the institutional and/or national research committee and with the 1964 Helsinki Declaration and its later amendments or comparable ethical standards.

Regarding the consent process for our study, we will obtain written consent from all participants. Participants will receive a Subject Information and Consent Form detailing the study’s purpose, procedures, risks, benefits, and their rights as participants. Additionally, participants were given a Participant’s Material Publication Consent Form to provide explicit consent for the publication of anonymized data or materials derived from their participation in the study. They will be given sufficient time to review the document and ask any questions before providing their signature indicating their voluntary participation. As our study did not involve minors, we did not need to obtain consent from parents or guardians.

### Trial registration

This study is registered in ClinicalTrials.gov under the registration number NCT05327153.

### Study design

This study will employ a double-blind, parallel-group randomized controlled trial to evaluate ACT’s effectiveness in promoting disease acceptance among breast cancer patients. This research protocol was developed in accordance with the SPIRIT (Standard Protocol Items: Recommendations for Interventional Trials) guidelines.

### Randomization and allocation concealment

Permuted block randomization with an allocation ratio of 1:1 is carried out. Participants will be randomly assigned based on odd (Group A) and even (Group B) rows using a computer-generated randomisation sequence. The randomization sequence, informing participant assignment to either intervention (Group A) or waitlist control (Group B) condition, is provided in opaque envelopes by one investigator not involved in participant recruitment or assessment. Blinded to the study assignment, participants will be allotted to either group in a 1:1 ratio. Participants will be randomly assigned to two groups: the intervention group receiving ACT intervention or the control group receiving standard treatment with non-therapeutic intervention.

### Blinding

Participants will be unaware of the study’s randomization into the designated groups, which will be carried out by a research assistant who is not otherwise involved. As a result, participants will not be informed of the group to which they are allocated.

Additionally, to minimize bias, the ACT intervention group and the control group will receive a 4-session program (with one hour per session, one session per week for 4 weeks). Participants in the control group will also receive equal attention (attentive listening) and time from the professional figure administering the program with non-therapeutic intervention(the participants in the control group are given equal duration of the session, number of sessions, duration of intervention and attended by the same therapists who conducted the ACT group. The participants will also be attended to with active listening by the therapists in the control group).

To maintain blinding throughout the study, the researchers will be blinded to participants’ randomized assignments into the groups. This process will be carried out by a research assistant who is not involved in the study. Additionally, data collection for this study will be conducted by the same research assistant, ensuring impartiality. The research assistant will remain unknown to the study’s hypotheses. Furthermore, data analysis for the study will be performed by statisticians who are not involved. The statistician will utilize a statistical analysis plan to conduct data analysis before the final unblinding of the data lock.

### Participants

Patients receiving treatment in oncology clinics at Universiti Sains Malaysia and Hospital Seberang Jaya will be recruited for the study. The criteria for study participation include: (1) diagnosed with breast cancer, (2) aged 18 years or older, (3) currently receiving treatment, (4) able to understand and communicate in the study language, and (5) willing to participate in the intervention and follow-up assessments. Patients are deemed ineligible for the study if they: (1) have severe cognitive impairment or significant psychiatric comorbidities (e.g., psychosis, substance abuse) that could hinder their participation in the study, (2) are diagnosed with malignant tumors, and (3) currently receiving any other form of psychological therapy intervention.

Sample size calculations will be based on power analysis using established effect sizes from previous studies on ACT interventions and disease acceptance among similar populations. We conducted an a priori power analysis using GPower 3.1.9.7 to calculate the estimated sample size needed for mixed ANOVA with control of three covariates: the type I error was 0.05, with a power of 0.95, three covariates, with two intervention groups and an effect size of 0.46 by referring to an RCT on the efficacy of ACT on illness cognition among breast cancer patients [28], Hence, the estimated sample size needed is 80 (with the inclusion of a 20% dropout rate). Hence, 40 subjects are required per group.

### Procedure

This research used a consecutive sampling technique for subject recruitment before randomly assigning them to the intervention and control groups. Potential participants will first be screened to determine their eligibility for study participation. Invitations to take part in this trial will follow. The investigator will ensure that participants receive a thorough explanation of the study goals and methods before agreeing to join the study. Participants will sign the Subject Information and Consent Form and Participant’s Material Publication Consent Form before enrolling in the research to indicate their agreement. We will ensure the participants’ anonymity and inform them about their right to withdraw from the study at any point, guaranteeing their confidentiality and autonomy. To ensure participants receive similar intervention, the trial adapts a brief ACT module by Shari et al. [31]. The therapist will administer the intervention when patients are in the hospital for treatment. The assessment batteries will be issued at three specific time points: before the intervention, immediately after the intervention, and three months following the intervention.

**Fig 1:**
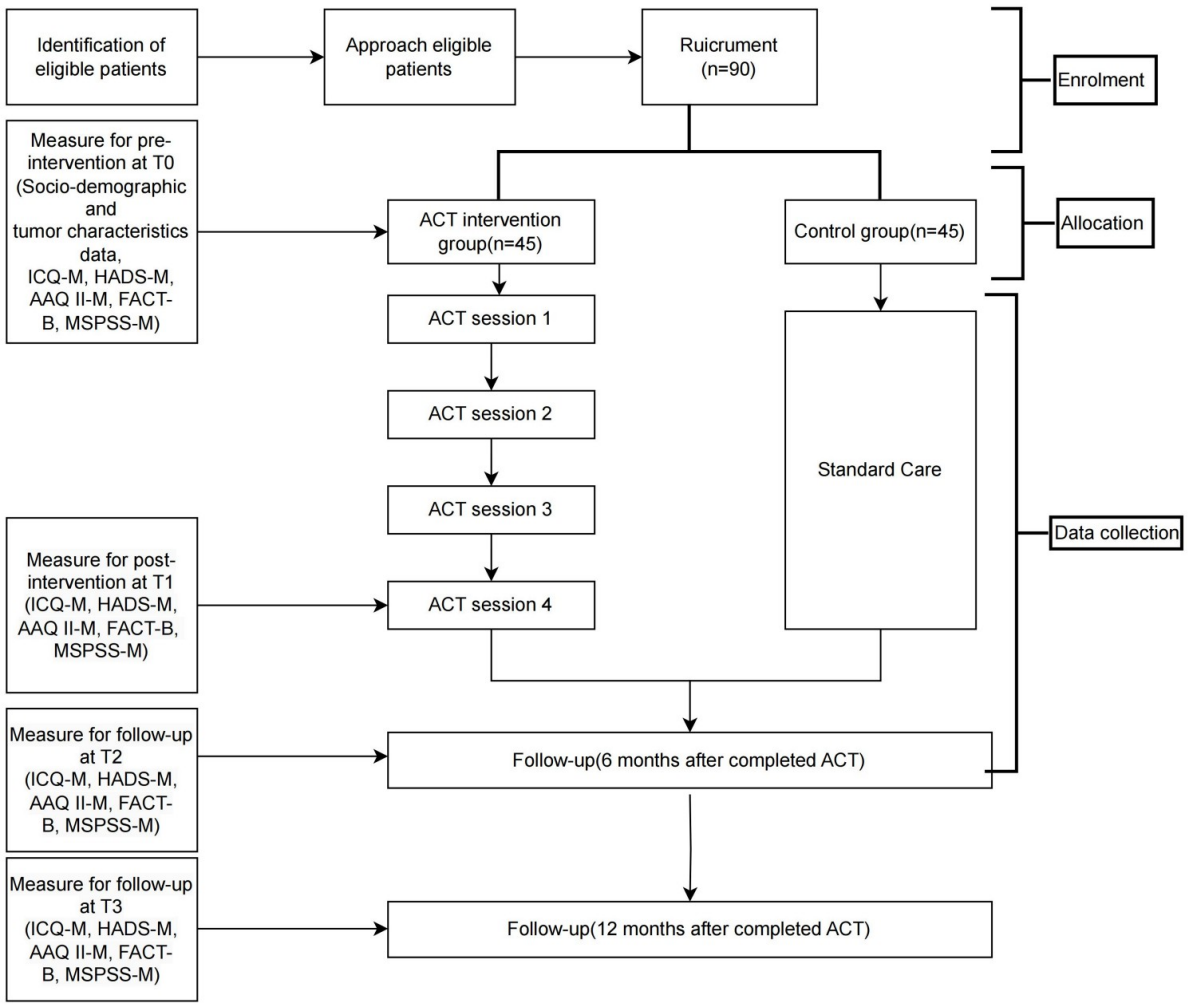
Scheduled assessment of the randomized control trial’s outcome measures.

**Fig 2:**
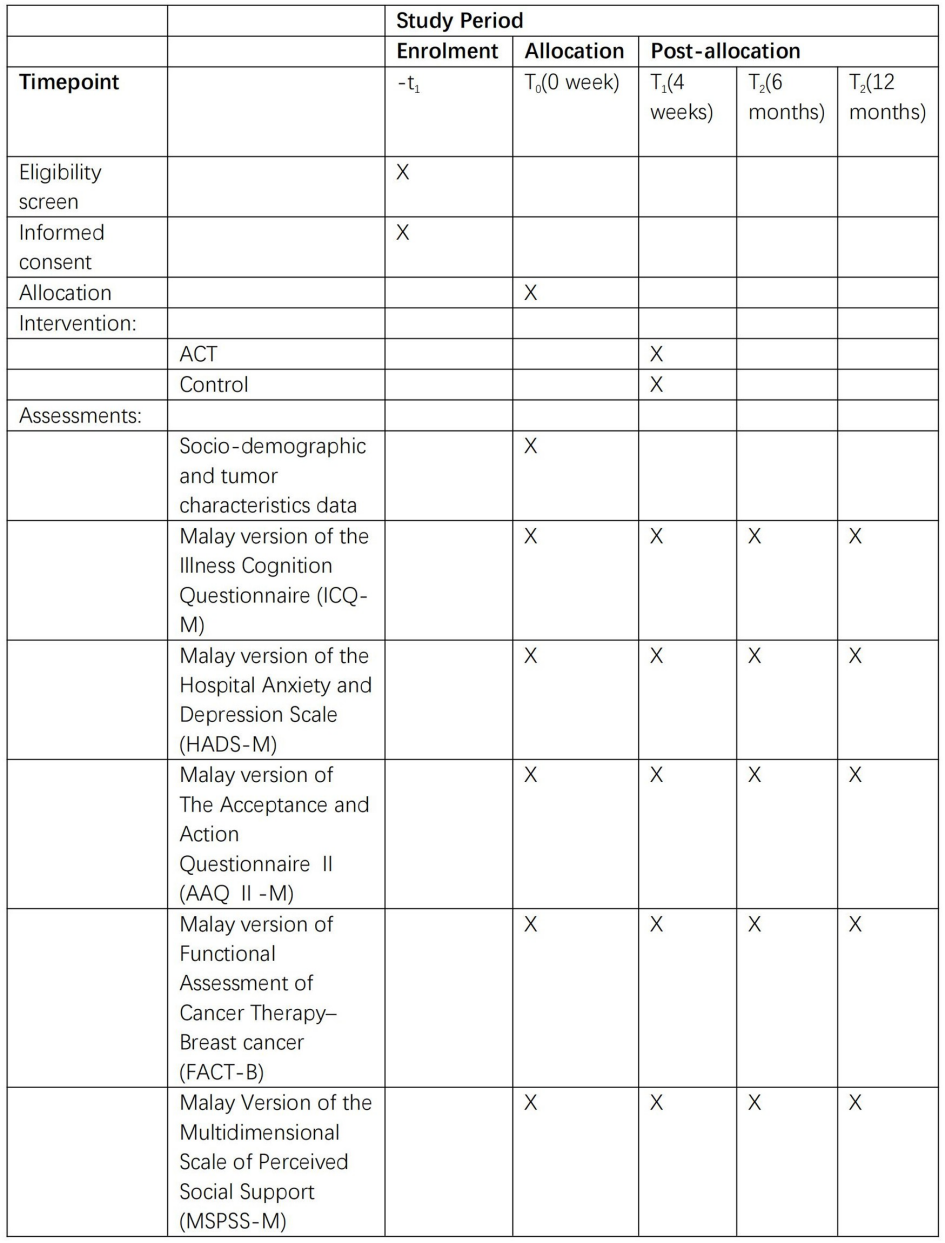
The flow of the research procedures and outcome measures.

Participants in the control group will receive the non-therapeutic approaches, such as reassurance, to allow patients to ventilate their problems and to conduct relaxation techniques such as deep breathing. The intervention and the control groups will receive a 4-session program to ensure ethical considerations and participant access to the intervention. The enrolment, interventions, and assessments of the study are summarized in Fig 1. Fig 2 shows the flow of the research procedures and outcome measures.

## Interventions

### ACT intervention

ACT is one of the most representative experiential behavior therapies in the third wave of cognitive and behavior therapy, called “contextual cognitive-behavioral therapy.” ACT aims to increase psychological flexibility so that people may dedicate themselves to a worthwhile and meaningful life via flexible therapeutic methods, as well as mindfulness, acceptance, defusion, self as context, value, and committed action. The interventions include evaluating cognitive fusion and values, accepting negative emotions and experiences, dissociating from personal experience, contact with the present and meditation training, determining importance, making behavior plans, etc. The ACT intervention is scheduled for 1 session per week for 4 weeks. Each session lasts for 1 hour. The activities in each session are summarized in Table 1. A detailed description of the activities in each session is presented in the S1 Appendix.

**Table 1:** Summary of content of ACT treatment sessions.

Session	Element in hexaflex	Content
Introductory	Therapeutic relationship, introduction to ACT	Establish a therapeutic relationship, conduct an intake interview (patient’s background, illness, coping mechanisms), develop a case formulation, and introduce ACT principles.
Let it go	Acceptance and defusion	Teach acceptance of unpleasant thoughts and emotions, use metaphors (e.g., tug-of-war with a monster) to practice cognitive defusion, reduce avoidance, and encourage observation of thoughts. Two home exercises on acceptance and defusion provided.
Show up	Contact with present moment, self-as-context	Mindfulness exercises to develop present-moment awareness and non-judgmental observation of thoughts, guide patients to view thoughts flexibly, and practice mindfulness exercises (e.g., mindful breathing).
Get moving	Values and committed action	Help patients identify values, set value-driven goals, guide action steps toward meaningful activities, and provide four home exercises (e.g., values assessment, mindfulness exercise).

### Control group

The control group participants will receive treatment as usual, including nonspecific and nontherapeutic approaches such as providing psychological understanding, identifying current problems, and practising deep breathing exercises. They will receive the same amount of time and attention from the same therapist as the intervention groups and participate in a one-session weekly program for four weeks.

### Treatment fidelity

The ACT intervention group will receive therapy administered by a therapist specifically trained in ACT. This therapist, a PhD student in psychiatry, is not otherwise involved in the study. They have received training from a licensed clinical psychologist with six years of experience in practising ACT. To ensure treatment integrity, independent monitoring will be conducted by a psychiatrist and a clinical psychologist trained in ACT, who will evaluate the delivery of interventions by the therapists in the ACT group. About 15% audio recordings of the psychotherapy sessions will be randomly selected and stratified according to the intervention phase (early, middle, or end). Then, treatment integrity assessments will be performed using (1) the Drexel University CT/ACT Therapist Adherence and (2) the Competence Rating Scale for the selected ACT session recording. Each treatment integrity assessor will assess half of the selected sessions. Then, the inter-rater reliability between the two treatment integrity assessors is computed to assess the treatment integrity of the therapists who are delivering the interventions. If the therapist is inconsistent in delivering some sessions, the therapist will discuss this issue with the principal investigator to solve any shortcomings during the interventions.

## Outcomes

Participants will be asked to complete self-report questionnaires in person or through secure online platforms. The questionnaires will cover various aspects, including demographic information such as age, race, educational background, marital status, and employment position. Disease-related information, such as the disease stage, current treatment, and other relevant factors, will also be gathered. Then, we need assessments of the primary and secondary outcomes. We use the electronic data capture system (EDC) to capture and store the data.

### Primary outcome: Disease acceptance

Disease acceptance will be assessed using the Malay version of the Illness Cognition Questionnaire (ICQ-M) for breast cancer patients. Evers created ICQ in 2001, and it assesses disease cognition from both positive and negative viewpoints, with three dimensions of helplessness, acceptance, and perception benefit. The better the condition of the related dimensions, the higher the score [32]. ICQ-M demonstrates strong psychometric properties, making it a reliable and acceptable tool for assessing disease acceptance among breast cancer patients. The ICQ-M exhibited good internal consistency, with Cronbach’s alpha values ranging from 0.742 to 0.927, indicating high reliability across its domains. Content validity was evaluated by a panel of experts who rated the relevance of each item, resulting in high item-level and scale-level content validity indices (I-CVI and S-CVI), affirming the appropriateness of the ICQ-M items. Construct validity was supported by factor analysis, which confirmed the structural integrity of the questionnaire, comprising 17 items distributed across two domains with good convergent and discriminant validity. Additionally, concurrent validity was evidenced by moderate correlations with the Acceptance and Action Questionnaire II (AAQ-II), indicating that the ICQ-M effectively measures the intended constructs in relation to similar tools. The ICQ-M’s reliability and validity make it suitable for measuring disease acceptance in Malay-speaking breast cancer patients [33].

### Secondary outcome

#### Psychological distress

To evaluate participants’ levels of anxiety and depression symptoms, assessments such as the Hospital Anxiety and Depression Scale (HADS) will be employed. The HADS, developed by Zigmond and Snaith, is a screening tool for non-psychotic anxiety and depression symptoms in hospitalized patients. It includes two anxiety and depression subscales [34]. In this study, we utilized the Malay version of the Hospital Anxiety and Depression Scale (HADS-M), translated by Yahya F. The HADS-M has shown excellent reliability, with a sensitivity of 90.0% and specificity of 86.2% for anxiety and a sensitivity of 93.2% and specificity of 90.8% for depression. These figures indicate that the HADS-M is a highly reliable tool for screening anxiety and depression in the Malaysian population. Additionally, the HADS-M has been validated in a Malaysian hospital setting and has proven to be a valid instrument for assessing psychological distress among patients. The scale’s psychometric properties, including its high sensitivity and specificity, affirm its acceptability and utility in clinical practice within Malaysia [35].

#### Quality of life

Physical condition, social and family situation, emotional state, functional condition, and a breast cancer-specific module make up the five dimensions of Functional Assessment of Cancer Therapy– Breast cancer (FACT-B). The FACT-B consists of 37 items: the higher the score, the better the overall survival treatment and the corresponding dimension [36]. The Malay version of the FACT-B is a reliable and valid tool for assessing the QoL in Malaysian breast cancer survivors. It demonstrated high internal consistency, with a Cronbach’s alpha of 0.88 for the overall scale and values ranging from 0.62 to 0.88 for individual domains. The FACT-B also exhibited strong concurrent validity, as evidenced by significant correlations with the Disabilities of Arms, Shoulders, and Hands (DASH) questionnaire and the Patient Health Questionnaire-Anxiety Depression Scale (PHQ-ADS). Additionally, the tool detected differences in physical, functional, and QoL measures between participants with and without lymphedema, confirming its known-group validity. These strong psychometric properties highlight the FACT-B’s suitability for use in clinical and research settings to evaluate the impact of breast cancer and its treatment on patients’ well-being in Malaysia [37].

#### Psychological inflexibility

The AAQ-II assessed psychological inflexibility or experiential avoidance. There are seven items in AAQ-II. A higher AAQ-II score suggests more psychological inflexibility [38]. The Malay version of the AAQ-II has demonstrated strong psychometric properties, making it a reliable and acceptable tool for assessing psychological inflexibility among Malay-speaking populations. It exhibited good content validity, reflecting the relevance and representativeness of its items. The internal consistency was high, with a Cronbach’s alpha value of 0.91, and the tool also showed excellent parallel reliability. Exploratory factor analysis confirmed that the AAQ-II Malay version is a unidimensional instrument. Adequate concurrent validity was established through significant correlations with other measures of similar constructs. Additionally, the sensitivity and specificity analyses indicated that the AAQ-II Malay version effectively distinguishes cancer patients with significant psychological inflexibility (scores above 17.5) from those without. These findings support the AAQ-II Malay version’s use in clinical and research settings to measure psychological inflexibility accurately [39].

#### Social support

The Malay version of the Multidimensional Scale of Perceived Social Support (MSPSS-M) for cancer patients covers three dimensions of social support (Family, Friends, and Significant Others). The MSPSS-M consists of 12 items, with higher scores indicating greater perceived social support by the patient. The MSPSS-M has demonstrated strong psychometric properties, making it a reliable and valid tool for assessing perceived social support among cancer patients in Malaysia. The MSPSS-M exhibited high internal consistency, with Cronbach’s alpha values ranging from 0.900 to 0.932, indicating excellent reliability. Confirmatory factor analysis supported the three-factor model of the original English version, confirming the construct validity of the MSPSS-M. Additionally, the MSPSS-M showed good convergent and discriminant validity, ensuring it accurately measures perceived social support and distinguishes it from unrelated constructs. The tool also demonstrated good content and face validity, indicating that the items are appropriate, comprehensive, and understandable for the target population. The MSPSS-M is a reliable and acceptable instrument for clinical and research settings to assess perceived social support among Malay-speaking cancer patients in Malaysia [40].

## Statistical analysis

### Descriptive analysis

Statistical software for social sciences (SPSS version 27) will be used to conduct data analysis. The study of sociodemographic data will make use of descriptive statistics. Race, age, marital status, educational background, work position, and illness state, including the stage at diagnosis, chemotherapy regimen, and time since diagnosis, are all described using frequency and percentage. Before implementing the ACT intervention, inference analysis will examine the significant differences between the waiting and intervention groups. The study’s primary analysis will adhere to the intention-to-treat (ITT) principle. Furthermore, the main effect will be accompanied by a 95% confidence interval. Statistical significance will be determined using a two-tailed approach and set at p<0.05.

To address any instances of missing data, the approach will be as follows: If missing data accounts for less than 5% of the total collected data, they will be disregarded. In cases where missing data exceed 5% but fall below 40% of the total collected data and are assumed to occur randomly, multiple imputations using restricted maximum likelihood estimation will be conducted using Stata 15. However, if missing data surpass 40% of the total collected data or are presumed to be non-random or entirely random, only the available data will be utilized for analysis in the study, and any absence of data will be acknowledged as a limitation in subsequent publications of the study’s findings [41].

### Mixed ANOVA analysis

The comparison of mean differences before and post-intervention will be examined to see whether any changes in illness cognition, anxiety, depression, psychological inflexibility, QoL, and social support among breast cancer patients in response to ACT intervention. Then, while controlling for confounders, mixed ANCOVA will be used to look at any significant changes in the measured variables between the waiting and intervention groups, with the group as the between-group factor and time as the within-group factor. A few confounding factors that may affect disease acceptance in cancer will be taken into account and controlled by using mixed ANCOVA, such as the level of pain and fatigue and monthly income [42, 43]. Age is also included as a covariance.

The effect sizes will be determined to evaluate how much patients’ perceptions of the measured variables changed with and without an ACT intervention. The formula of Cohen’s d (Rosnow Rosenthal, 1996) [44] is used to calculate the effect size. According to Cohen (1988), the effect size will be considered small if (d = 0.2), medium (d = 0.5), and large (d = 0.8).

### Mediators and moderators

The mediation analysis will be conducted using the PROCESS macro-Version 3.5 developed by Andrew F. Hayes. This research uses a single mediator model to examine the total, direct, and indirect effects. The independent variables will be the groups in the RCT study (ACT intervention group and control group). At the same time, the dependent outcomes will be the change scores of illness cognition and QoL from pre-treatment to post-assessment. The change score is chosen as the dependent variable to assess the improvement of scores between pre-and post-treatment. Proposed mediator variables include psychological inflexibilities, anxiety, depression and social support. PROCESS will be used to validate the mediating effect of psychological inflexibility and disease cognition on the relationship between the ACT intervention and QoL. The Bootstrap program will confirm the direct and indirect effects. Additionally, social support will be introduced as a moderating variable in the coping path to create a final model that verifies the overall mediating product.

## Safety monitoring

Ensuring participant safety is of the utmost importance throughout the study. Therapists delivering the ACT intervention will undergo training to promptly identify and address any potential adverse events or reactions that may arise during therapy sessions. In the event of an adverse incident, participants will have the option to withdraw from the research. Participants will be actively encouraged to report any concerns or negative experiences related to the intervention. Any negative medical development affecting a trial participant unrelated to the treatment intervention is considered an adverse event (AE). Any unwelcome or unexpected indications, symptoms, or transient diseases are included, regardless of how they relate to the experimental intervention.

Participants are strongly advised to regularly contact the research team by phone and quickly report any adverse events (AEs) they may encounter. Participants will get a study card with the research team’s contact details. If an adverse event (AE) does take place, it will be noted in the “Adverse Event” part of the case report form (CRF) for the research, and, if required, a thorough adverse event report will be created. The name of the AE, its start and recovery dates, intensity, relevance to the intervention under study, measures done in response to the intervention, any therapy given, and the result (whether resolved or continuing) are all included in these reports. At Universiti Sains Malaysia, the Human Research Ethics Committee will be informed of severe adverse occurrences. The Common Terminology Criteria for Adverse Events (CTCAE) version 5.0 will be used to monitor the occurrence of adverse effects among participants.

## Data sharing and dissemination

Authorship eligibility will be determined by suggestions made by the International Committee of Medical Journal Editors. Access to the study’s files for data analysis or publishing is only allowed for the study’s corresponding author and co-authors. The study findings will be disseminated through various channels to maximize their impact and reach. Data collected from the survey will be protected and stored securely. Only aggregated and de-identified data will be reported in publications, ensuring the anonymity of participants. The results will be analyzed and prepared for publication in peer-reviewed journals, and presentations will be made at scientific conferences and meetings relevant to breast cancer, psycho-oncology, and psychological interventions. In addition, efforts will be made to communicate the findings to appropriate stakeholders, including healthcare professionals, patient advocacy groups, and policymakers, to inform clinical practice and potential implementation of ACT interventions for breast cancer patients. The researchers of this study all state that they have no conflicting financial or other interests.

## Limitations and future research

It is important to recognise certain potential limitations of the study. Firstly, the study design relies on self-report measures, which are susceptible to response biases. To address this, participants will be assured of the confidentiality and anonymity of their responses to encourage honest reporting. Secondly, the study will be conducted in Eastern Malaysia and may not fully capture the diverse experiences of breast cancer patients nationwide. Future research should replicate the findings in larger, more diverse populations to enhance generalizability. Third, the sample size may be relatively small. However, it is deemed sufficient upon sample size calculation, considering the heterogeneity of the participants and the self-reported measuring tools used in the study, which may further affect the level of generalisation. Finally, this study did not assess the survival rate of breast cancer patients as an outcome.

In summary, this study is committed to maintaining the highest ethical standards and prioritizing participant safety while examining the impact of ACT on disease acceptance among breast cancer patients.

## Discussion

Breast cancer is a significant global health concern affecting numerous individuals. ACT is crucial in fostering disease acceptance and improving QoL among breast cancer patients. This research protocol uses a randomized controlled design; participants are fairly assigned to either the ACT intervention or control group, minimizing confounding variables and enhancing the study’s reliability. We hypothesize that this study could significantly impact treatment outcomes by showing that ACT increases patient disease acceptance and improves QoL, potentially making ACT a beneficial addition to traditional medical treatment. This approach may help patients adjust to their diagnosis and treatment challenges, enhancing their QoL and treatment adherence. This study will also indicate the mechanism underlying the efficacy of ACT in improving QoL of breast cancer patients, which may be mediated by illness acceptance and psychological flexibility, indicating the importance of enhancing these psychological traits. ACT is one of the psychological treatments that could enhance these traits in cancer patients. The comprehensive methodology may also act as a methodology framework to allow replication of this study to evaluate the efficacy of other psychological treatments to enhance the QoL via psychological inflexibility and disease acceptance.

However, this study’s limitations include a small sample size, which may affect the generalizability of the results, and the risk of bias due to conducting the study within a single nation. Future research should reproduce this study with a larger and more diverse participant cohort to improve the generalizability of the findings and provide a more thorough assessment of ACT’s efficacy.

In conclusion, this research protocol examines how ACT affects disease acceptance and QoL in breast cancer patients. ACT could be included in routine treatment, improving coping mechanisms and mental health if effective. Further studies are needed to confirm these results and explore the long-term impact of ACT on disease acceptance in a broader population.

## Supporting information

S1 Appendix A detailed description of the activities in each ACT intervention session. (DOCX)

S1 Checklist SPIRIT 2013 checklist: Recommended items to address in a clinical trial protocol and related documents*. (DOC)

S1 File (DOCX)
