# Supplementary material for: Effectiveness of Acceptance and Commitment Therapy (ACT) on disease acceptance for breast cancer patients: Study protocol of a randomized controlled trial
Source: PLoS One. 2024 Nov 11;19(11):e0312669. doi: 10.1371/journal.pone.0312669 (PMC11554193; doi:10.1371/journal.pone.0312669)
Supplement: S1 Appendix — (DOCX) [file pone.0312669.s001.docx]

**S1 appendix: The detail description of the activities in each ACT intervention session**

In the first session, we establish a therapeutic relationship. To build trust, therapists

introduce themselves and their training. After that, we have an intake interview when

participants provide personal information about themselves, including address, age, and

experiences with their condition, including changes in body image and coping

techniques. We map the respondent’s difficulties and formulate a case using this

information. Lastly, we showcase our intervention project and discuss how ACT may be

used to regulate negative thoughts and emotions from various angles.

For the second session, titled “Let it Go,” we focus on Acceptance-Creative

Hopelessness. This approach helps patients accept unavoidable realities rather than

trying to escape them. When faced with negative experiences or unpleasant thoughts,

allowing space for these feelings is crucial instead of trying to control them. The goal is to interact with these experiences without resistance. We use metaphors like a

tug-of-war with a monster or passengers on a bus to help patients accept and engage

with their emotions non-judgmentally. Patients are encouraged to face and accept

physical changes and dysfunction after surgery rather than avoiding or fighting them.

Patients receive home practice worksheets for acceptance and defusion techniques to

support this.

For the third session, ”Show up,” we focus on self-as-context, the aspect of self that

observes thoughts and emotions without becoming entangled in them. This ”observing

self” remains constant throughout life’s changes. Patients learn to recognize this part of

themselves while observing their thoughts and feelings with curiosity and openness.

Mindfulness exercises, such as emotional storm, dropping anchor, and chessboard, help

patients observe their emotions without judgment and reduce fear and worry. The

session ends with guidance on practicing mindfulness through mindful breathing, eating,

and bathing.

For the fourth session, “Get moving,” patients identify their values and set goals

aligned with those values. They are informed about advances in breast cancer diagnosis,

treatment, and prognosis to help adjust their mindset and restore confidence in life.

Patients are urged to evaluate the meaning of life and choose their course for the future.

Activities like the bullseye, compass, and values evaluation help children make

incremental progress toward living a life that is meaningful. The four at-home activities

offered are a values assessment, an action list, a mindfulness exercise, and a values list.

Therefore, the brief ACT therapy will be delivered in an individual setting. A brief

ACT module by Shari et al. inspired the ACT module. The intervention was divided

into three modules, each including an essential element of ACT [31]. Each module is

made up of two hexaflex elements. The modules will be covered in 4 sessions: 1 hour

each.
