# Supplementary material for: Effectiveness of Acceptance and Commitment Therapy (ACT) on disease acceptance for breast cancer patients: Study protocol of a randomized controlled trial
Source: PLoS One. 2024 Nov 11;19(11):e0312669. doi: 10.1371/journal.pone.0312669 (PMC11554193; doi:10.1371/journal.pone.0312669)
Supplement: S1 File — (DOCX) [file pone.0312669.s003.docx]

**Supplementary appendix: Research protocol approved by the Human Research Ethics Committee of Universiti Sains Malaysia**

**Research title:** The efficacy of Acceptance and Commitment Therapy (ACT) on disease acceptance and quality of life in breast cancer patients: A randomized controlled trial

**Principal investigator (MMC No. if applicable):** Song Wenjun

**Co-researchers: (MMC No. if applicable):** Nor Shuhada Binti Murad, Dr. Mohammad Farris Iman Leong B Abdullah, Dr. Nurul Izzah Binti Shari, Noor Mastura Binti Mohd Mujar, Dr. Lu Ping

**Introduction**

*According to the World Health Organization (WHO), by 2020, there will be 2,179,457 new instances of breast cancer in women worldwide, accounting for 24% of all new cancer cases in women. Breast cancer, on the other hand, has a favorable prognosis and a lengthy survival span. (Cancer Research UK: Breast Cancer Survival Statistics., n.d.). The five-year standardized relative survival rate for breast cancer was 85.0 %, while the one-, five-, and ten-year observed survival rates were 95.8%, 85.0 %, and 75.9%, respectively (Cancer Research UK: Breast Cancer Survival Statistics., n.d.). Breast cancer has a high incidence, but its survival rate is generally greater than that of most other cancers, which means that long-term survival rates are improving, but most patients' quality of life is poor (Duijts et al. 2011). Malignant tumors are often linked to death in the minds of patients. Patients are under a great deal of psychological stress while dealing with the physical discomfort caused by the condition. Anxiety and despair are common bad feelings (Carroll et al. 1993). The psychological state of cancer patients will be influenced by the diagnosis, complications, and treatment side effects (Mehnert et al. 2014). Breast cancer patients had a greater rate of mental disorders after diagnosis than the general population, with 42 percent meeting rigorous diagnostic criteria for mental disorders within four weeks of diagnosis, the highest rate of all cancers (Mitchell et al. 2008). Breast cancer patients are more likely to experience subclinical psychological illness and symptomatic psychological discomfort, with a frequency of 30% to 75% (Andrzej Nowicki & Zaneta Ostrowska, 2008). In the realm of oncology, it's critical to figure out how to improve the psychological rehabilitation of breast cancer patients.*

*According to a recent study, 20% of breast cancer patients following mastectomy still struggle with acceptance of their diagnosis (Eifert & Heffner, 2003). According to empirical research on acceptance intervention, participants who were exposed to aversive interoceptive stimuli and underwent acceptance intervention had decreased behavioral avoidance, strong dread and cognitive panic symptoms, and catastrophic thoughts (J G L A-Tjak et al. 2015). Individual anxiety levels can be effectively reduced by good acceptance (Diego et al. 2016). Furthermore, as a result of the shift in the modern medical model, breast cancer treatment now focuses not only on extending patients' survival times and lowering mortality but also on improving their quality of life (Mokhatri-Hesari & Montazeri, 2020). However, while surgery is one of the most essential therapies for breast cancer, the loss of breast tissue produced by surgery will compromise the integrity of a woman's body and alter her appearance to some extent. Patients with breast cancer have a lower body image after surgery than healthy persons, which lowers their quality of life. (Bakht & Najafi, 2010). Experiential avoidance is a characteristic that influences body image, and researchers have found that minimizing experiential avoidance practices can improve body image. (Bakht & Najafi, 2010). Meanwhile, social support can encourage breast cancer patients to utilize positive coping strategies, increase their cognitive understanding of the disease, boost their self-esteem, and therefore improve their quality of life (Janowski et al. 2020). The most important aspect of the adaptation process is accepting the sickness. Better sickness acceptance can lead to less stress and higher self-esteem, which makes it easier to adjust to new health status. As a result, the goal of this study is to see if boosting illness acceptance improves the physical and psychological well-being of breast cancer patients.*

**Problem statement & Study rationale**

*Studies have shown that psychotherapy can effectively improve the psychological pain of cancer patients, relieve anxiety and depression, and improve quality of life (Tao et al. 2015). With the development of psycho-oncology, psychological intervention is increasingly used in patients with breast cancer (Hsu et al. 2010a; Smith et al. 2021; Speck et al. 2009). This study uses ACT intervention to improve mental health and quality of life in breast cancer patients. In a pilot study, Jing Han et al (2019) found that ACT intervention was effective in improving psychological flexibility, illness cognition (including acceptance), and quality of life (Han et al. 2019). Nevertheless, there is no randomized controlled trial conducted to confirm the findings of this preliminary study. So, the purpose of this research was to fill this gap and it also aimed to provide new insights into the relationship between disease acceptance and the quality of life in patients with breast cancer. Despite the convincing evidence on the relationship between breast cancer patients with psychological flexibility, depression, body image, and quality of life, the mechanism remains unclear. As a result, this study aims to elucidate the role of psychological inflexibility, illness cognition, and body image as mediators in the mechanism through which ACT improves the quality of life among breast cancer patients. The findings of mediation analysis will help to establish the effectiveness of ACT therapy. What is more, social support can encourage patients to use positive coping policies and reduce the use of negative policies. Arora et al followed 103 postoperative breast cancer patients and gave them supportive intervention. The results showed that during the treatment, the patient's body image, physical and mental health, and the soundness of various functions were enhanced (Horwitz et al. 2001). Therefore, in our study social support will be a moderator variable to illustrate the change in illness cognition, body image, psychological flexibility, depression, anxiety, and quality of life according to ACT intervention, thus further elucidating the mechanism of change. Overall, the findings of mediation and moderation analysis will help to extend the knowledge, expand the theories, and strengthen the effectiveness of ACT therapy according to the patient’s needs. Furthermore, positive social support and effective psychological intervention can reduce the physical and mental symptoms of breast cancer patients to a certain extent, at the same time improving the quality of life and improving mood of breast cancer patients is also related to the whole treatment effect of breast cancer patients.*

**Research Question(s)**

**Study I**

1. *Is the Malay version of the Body Image Scale reliable and valid for cancer patients?*
2. *Is the Malay Illness Cognition Questionnaire reliable and valid for cancer patients?*

**Study II**

*1. What are the changes in psychological flexibility, disease cognition, anxiety and depression, and quality of life in patients with breast cancer after ACT intervention?*

*2. What is the mechanism that changes the anxiety, depression, and quality of life of breast cancer patients after ACT intervention?*

1. *What is the effect of social support as a moderator variable on disease acceptance and quality of life during ACT intervention?*

**Objective**

**General:**

*The overarching objective of the study is to investigate the effect of ACT intervention on the quality of life of patients with breast cancer.*

**Specific:**

**Study I**

*1. To test the reliability and validity of the Malay version of the Body Image Scale (BIS) in cancer patients.*

*2. To test the reliability and validity of the Malay version of the Illness Cognition Questionnaire (ICQ) in cancer patients.*

**Study II**

*1. To examine the efficacy of brief acceptance and commitment therapy on disease acceptance, psychological flexibility, anxiety, depression, quality of life, and perceived social support of breast cancer patients.*

*2. To explore the mechanism between acceptance and commitment therapy with disease acceptance and quality of life in breast cancer patients.*

*a) To explore the mediating role of psychological flexibility and disease acceptance in response to ACT intervention toward the changes in anxiety, depression, body image, and quality of life in breast cancer patients.*

*b) The role of social support as a moderator in the process of the efficacy of ACT on disease acceptance and quality of life in breast cancer patients.*

**Literature review**

*1. Illness cognition in breast cancer*

*1.1 Factors associated with disease acceptance/illness cognition*

*A study of 313 women with breast cancer found that having no children, not returning to work, having no medical insurance, treatment status, and disease duration all influenced disease acceptance. Receiving continuous therapy, not having children, and not returning to work were all positively related to helplessness and negatively associated with acceptance, whereas age was both negatively and favorably associated with helplessness and acceptance. Perceived advantages were adversely correlated with continued therapy and the lack of medical insurance. Furthermore, the length of a patient's illness was linked to their willingness to accept it (Han et al. 2018). During chemotherapy, patients frequently have side effects such as nausea, vomiting, and hair loss, whereas those undergoing mastectomy typically experience significant psychological changes (Aerts et al. 2014), which may lead to negative disease cognition and lower levels of acceptance. Furthermore, whether or not metastases have been diagnosed is the most important socioeconomic determinant influencing disease acceptance [21].*

*Finally, medical personnel should pay special attention to high-risk patients with poor cognitive conditions, such as young female patients, those without health insurance, and those who are currently receiving treatment (Han et al. 2018).*

*1.2 Emotion and acceptance/illness cognition*

*Breast cancer diagnosis and treatment will cause significant emotional distress throughout the treatment and rehabilitation process (Ludwigson et al. 2020). The National Cancer Centre Network (NCCN) defines "emotional distress" as an unpleasant emotional experience with many factors (Riba et al. 2019) which can seriously damage the mental health of patients [24]. According to studies published in other countries, 20 percent to 38 percent of female breast cancer patients have severe psychological distress within a year of receiving their diagnosis (Irvine et al. 1991). Female breast cancer patients are 1.5 percent to 46 percent likely to be depressed (Massie 2004). It's also been observed that both surgical therapy for breast cancer and the disease itself can lead to depression, with rates ranging from 3% to 55% (Miller and le Breton-Miller 2005). Patients' fears about tumors, whether or not they can acquire matching social support, economic strain, different forms of surgery and follow-up treatment plans, and so on are all factors that contribute to these negative psychological pressures. As a result, having an objective understanding of the sickness and actively accepting the disease can help to decrease psychological stress. Case-control research, on the other hand, found that breast cancer patients with low disease acceptance* had higher levels of despair and anxiety (Vespa et al. 2012).

*1.3 Quality of life and acceptance/illness cognition*

*The survival time of breast cancer patients has improved significantly as surgery, radiotherapy, and chemotherapy have progressed. However, mastectomy, radiotherapy, and chemotherapy, as well as adverse reactions, have a significant impact on the physical, psychological, social, and overall quality of life of breast cancer patients. As a result, breast cancer patient’s quality of life is poor to moderate (Lee et al. 2012). According to Leak's findings, physical and psychological stress hurt breast cancer survivors' quality of life (Leak, Hu, and King 2008). As a result, enhancing the quality of life for breast cancer patients is critical. Positive disease acceptance/illness cognition in breast cancer patients has been proven to not only improve quality of life but also diminish experience avoidance behavior, according to the study (Han et al. 2021).*

*1.4 Body-image and acceptance/illness cognition*

*Body image is a multi-dimensional and dynamic structure that includes emotional expression, imitation, identification, attractiveness, and social interaction. It refers to the image of one's own body produced in one's head, that is, how to treat one's own body (Han et al. 2021). Chemotherapy-induced baldness, peripheral neuritis, and amenorrhea in breast cancer patients, while radiotherapy caused redness and sensitivity of the skin around the breast; these therapeutic connections can contribute to alterations in female body image (S. C. Chen et al. 2018; Kowalczyk et al. 2019). Body image disorder encompasses not just unhappiness, but also the concepts of cognition and behavior, as well as body image-related cognitive alterations and avoidance behavior Fingeret, Teo, and Epner 2014). In conclusion, most of the studies indicated that body image disorder gave a significant impact on depression and anxiety (Chang et al. 2019), self-esteem (Hernández et al. 2015), and social avoidance (Favez et al. 2016). Positive coping styles and a reasonable disease assessment, on the other hand, can predict body image and lower the probability of cancer death (Cousson-Gélie et al. 2007). Furthermore, the patient's body image regulation is more satisfied with positive disease acceptance, but negative coping styles worsen the individual's stress response, which is harmful to the patient's physical and mental health (Pikler and Winterowd 2003).*

*2 Interventions that encourage disease acceptance/illness cognition in breast cancer patients*

*2.1 Cognitive behavioral therapy (CBT)*

*CBT emphasizes changing the content of an individual’s unreasonable cognitions during treatment. Cognitive behaviour group therapy (CBGT) for patients with breast cancer includes two parts: cognitive remodelling and behaviour training. The researchers adjusted the content of cognitive remodelling and conducted targeted behavior training according to the purpose of the study. CBGT is used to relieve subclinical mood disorders in patients with breast cancer and to control or relieve disease-related symptoms. Stagl et al reported intervention in a clinical randomized controlled trial of cognitive-behavior stress management (CBSM) in breast cancer patients. 11-year follow-up found that CBSM had long-term positive psychosocial effects on early breast cancer patients. In this study, 240 postoperative patients with early breast cancer were randomly divided into a 10-week CBSM intervention group and a 1-day psychological education control group. The intervention included cognitive remodelling, coping strategy training, self-confidence training, emotional management, and relaxation training. After 6 months, 12 months, 5 years, and 11 years after the intervention, a depression questionnaire survey was conducted for patients with breast cancer, and it was found that the scores in the intervention group were better than those in the control group at each time point (Stagl et al. 2015).*

*Studies have found that CBGT can prevent breast cancer recurrence and improve survival (Fawzy, Canada, and Fawzy 2003), (Andersen et al. 2008). But Kissane’s study found that CBT could not prolong the survival time of breast cancer patients (Kissane et al. 2004). Cognitive-behavioural therapy with cognitive reassessment as the core is not omnipotent in psychotherapy. Unlike traditional CBT, ACT uses a cross-diagnostic treatment model.*

*2.2 ‘Third Wave’ Approaches*

*Acceptance and commitment therapy (ACT) is one of the most representative experiential behaviour therapies in the third wave of Cognitive and Behaviour Therapy, named “contextual cognitive-behavioural therapy”. ACT aims to enhance psychological flexibility to help individuals devote themselves to a valuable and meaningful life, with mindfulness, acceptance, defusion, self as context, value, and committed action as well as flexible treatment techniques. Mohabbat-Bahar applied ACT group intervention to breast cancer patients, focusing on solving patients' anxiety and depression. In this study, 30 patients with breast cancer were randomly divided into intervention groups and control groups by a convenient sampling method. The interventions include evaluating cognitive fusion and values, accepting negative emotions and experiences, dissociating from personal experience, contact with the present and meditation training, determining values, making behaviour plans, and so on. Patients in the control group received routine nursing. The study found that ACT group intervention can reduce anxiety and depression scores in patients with breast* cancer (Moradi-Joo et al. n.d.).

*3 ACT*

*3.1* *Theoretical foundations of ACT*

*The theoretical foundation of ACT is Relational Frame Theory (RFT) based on the functional contextualism philosophy, which suggests that the main psychological problem is derived from verbal and cognitive interaction with the environment, leading to behaviours contrary to long-term values and psychological inflexibility. The psychopathological model of ACT includes six core parts: experiential avoidance, cognitive fusion, attachment to the conceptualized self, conceptualized past and feared future, lack of value clarity, and inaction. Experiential avoidance in the ACT pathological model means that individuals avoid contact with their specific experiences and make behaviour to change the form, frequency, and related situations of these experiences (Hayes et al. 1996).*

*According to RFT, experiential avoidance brings pain because it is based on human language and cognition, therefore human beings are unable to get rid of the pain by avoiding past painful situations and related factors. The framework of relationships established by human beings through language will make pain inevitable (Hayes 2016).*

*3.2 Psychological inflexibility*

*3.2.1 Cognitive fusion*

*Cognitive fusion means that people will associate adverse emotional reactions with accidental events and environment and immerse themselves in thinking, language, or evaluation, which is one of the important indicators of psychological flexibility (Jacqueline G.L. A-Tjak et al. 2015), the stronger the cognitive fusion, the lower the psychological flexibility. Cognitive fusion is one of the six core processes of Acceptance and Commitment Therapy. It describes a process where a person becomes excessively entangled in their thoughts, such that these thoughts dominate behaviour. The illness cognition of cancer patients produces anxiety through the mediated role of cognitive fusion (Gillanders et al. 2015).*

*3.2.2 Experiential avoidance*

*Newly diagnosed women cannot accept the “patient” identity (Taleghani, Yekta, and Nasrabadi 2006), so they deny the fact of diagnosis. Denial of the diagnosis is a psychological protective mechanism that buffers the influence of bad news, if patients do not experience denial, they may become intolerable, and sometimes impulsive, and engage in negative behavior (Zhang et al. 2017). A survey of 115 cancer patients by Seo in Japan shows that cancer patients will show shock and emotional changes after learning the diagnosis, but most patients recover after learning the diagnosis for some time. However, the psychological impact can last for a long time (Group psychotherapy for recently diagnosed breast cancer patients: a multicenter feasibility study - Spiegel - 1999 - Psycho-Oncology - Wiley Online Library n.d.). Naturally, denial that lasts for too long may result in late treatment because these patients may need to recheck their diagnosis again and again (Zhang et al. 2017). Individuals are prone to repressed thoughts in their natural state (Wang, Hagger, and Chatzisarantis 2020). Eskelinen et.al believed that unwillingness to express personal emotion or emotional depression is a characteristic of breast cancer (ESKELINEN and OLLONEN 2011). However, suppressing pain does not reduce the painful experience (Cioffi and Holloway 1993). That is to say, acceptance does not mean tolerance.*

*3.3 The core processes of ACT*

*3.3.1 Acceptance*

*Acceptance is the opposite of cognitive fusion. Acceptance refers not only to tolerance but also to a positive rather than critical accommodation of past events and present experiences, that is, to make room for painful feelings, impulses, and emotions. Not to resist, control and escape them, but to observe them as objects (Jacqueline G.L. A-Tjak et al. 2015). It has been reported that the individual can identify naturally generated negative thoughts and bring contradicted evidence against those thoughts to subsequently help the individual to interpret the situation in a more adaptive way, which constitutes the active cognitive restructuring process (Zhang et al. 2017). So, the patient can progress acceptance of the disease.*

*Studies have shown that the acceptance process included five stages: non-acceptance, passive acceptance, willingness to accept, behavioral acceptance, and transcendence of acceptance (S. Q. Chen et al. 2017). Other researchers suggested that acceptance is a developing process that carries emotional and behavioral responses and has both passive and positive states. Active acceptance was found to involve patients fighting breast cancer actively with motivations such as the love of their families and being patient and willing to tolerate the disease. Whereas passive acceptance of the disease was found to occur when women are trying to be satisfied with their present condition and think perhaps that something worse could be happened (Taleghani, Yekta, and Nasrabadi 2006). Women who passively accepted the disease were at significant risk of poor long-term psychological adjustment (Hack and Degner 2004). Horgan and colleagues explored the process by which 20 patients with breast cancer underwent positive psychological changes following their diagnosis. In this study, the acceptance by the women of having breast cancer appeared to be a prerequisite for their ability to effectively manage their illness (Horgan, Holcombe, and Salmon 2011). In addition, the results from the study reported that acceptance is adaptive with the treatment stage (Roussi et al. 2007) and provides an in-depth exploration into accepting having breast cancer among women. Overall, acceptance-based coping was found to be one theme of coping psychologically with the disease and predicted less psychological distress and depression (Jensen et al. 2014).*

*3.3.2 Defusion*

*Defusion refers to the ability to separate or detach from painful thoughts, and emotions (Harris 2009). For example, individuals can learn to notice or observe their thoughts as they occur, without judgment, rather than becoming entangled in them. They can also learn to see their thoughts as words or pictures rather than as truths. Cognitive defusion aims to alter how individuals relate to/interact with unwanted private experiences by developing environments/contexts wherein their unhelpful functions are reduced (Hayes et al. 2006).*

*3.3.3 Contact with the present moment*

*ACT allows patients to come into contact with the present through mindfulness acceptance, accepts situations that lead to internal experience, and reduces the control and adjustment of their own internal experience (Twohig 2012). The mindfulness-awareness exercise was applied to postmenopausal young breast cancer patients and its effect was discussed. The results showed that after the intervention, the symptoms such as stress perception, pro-inflammatory signal, fatigue, and sleep interruption decreased, and the positive feelings such as the meaning of life increased (Bower et al. 2015).*

*3.3.4 Self-as-context*

*There are thought to be two distinct parts to the self, the ‘thinking self and the ‘observing self’ (Harris 2009). The former generates thoughts, fantasies, plans, etc. Whereas the observing self (or the self-as-context) allows us to be aware of what we are thinking, feeling, etc. at any moment. Within self-as-context, an individual is no longer defined by the content of their private experiences and so difficult situations, as well as the verbal evaluations they give rise to, can become less disabling/threatening (Flaxman, Blackledge, and Bond n.d.). During ACT the observing self is encouraged through experiential and mindfulness activities, as well as metaphors (Hayes et al. 2006).*

*3.3.5 Values*

*Values can be defined as chosen qualities of purposive action that can never be obtained as an object but can be instantiated moment by moment (Hayes et al. 2006). That is values describe how we wish to act continually and provide direction for life (Hayes et al. 2006).*

*3.3. Committed action*

*ACT encourages individuals to put their values into action (Flaxman, Blackledge, and Bond n.d.). That is ACT cultivates increasingly larger patterns of effective action connected to one’s chosen values (Hayes et al. 2006). In this way, ACT appears similar to traditional behavioral therapy. Values can never be obtained as an object. However, concrete (behavior change) goals linked to one’s values can be worked towards (Hayes et al. 2006) using a range of behavioral interventions (Harris 2009).*

*4 ACT on breast cancer patients*

*4.1 Intervention of ACT in different stages of breast cancer*

*Patients with breast cancer face psychological discomfort from the moment they are diagnosed. Longitudinal studies have found that the level of distress after a diagnosis is higher than the level of distress at a later time. (Jones et al. 2015). Patients may experience clinical anxiety and depression before surgery, particularly in elderly women and women with lower levels of education. (Civilotti et al. 2021). After a mastectomy, patients frequently experience quick psychological and social changes as well as strain (Krok, Baker, and McMillan 2013). During adjuvant chemotherapy, the patient may experience anxiety and depression. (Gan et al. 2019; Silva, Zandonade, and Amorim 2017). Anxiety symptoms are common in people with newly diagnosed metastatic breast cancer, while depression is uncommon. (Park et al. 2018). Breast cancer patients in stages I and II usually have a better prognosis and a longer survival period (W. Chen et al. 2016), whereas patients diagnosed with stage Ⅳ breast cancer have a higher risk of depression and anxiety disorders (Tsaras et al. 2018).*

*An ACT intervention after breast cancer surgery was found to be useful in reducing postoperative pain and anxiety in one trial. (Hadlandsmyth et al. 2019). Another feasibility study of a six-session telephone-based ACT intervention for symptom interference in patients with metastatic breast cancer indicated that fatigue and sleep interference was reduced. (Mosher et al. 2020). ACT can increase mental flexibility, disease awareness, and quality of life in patients with breast cancer who are in the early phases of chemotherapy, according to a study (Han et al. 2019).*

*Overall, interventions aimed at reducing pain during these vulnerable periods can help patients recover, and the weeks following surgery are a good time to start, as this is when stress and immune function are at their highest. (Witek-Janusek et al. 2008).*

*4.2 ACT Intervention on disease acceptance*

*Acceptance is one of the most popular coping strategies for breast cancer sufferers. (Stanton, Danoff-burg, and Huggins 2002). It can forecast the reduction of psychological pain and has a long-term good adjustment effect on patients' mental health (Carver 1997) and negative emotions (Elumelu, Asuzu, and Akin-Odanye 2015). Previous breast cancer intervention trials have primarily focused on the acceptance of one's body image (Hsu et al. 2010b). Patients' acceptance of the disease encompasses both factual and societal acceptance, in addition to body image. Patients’ favorable perceptions of the disease improved mental health, and positive and worthwhile behavior activities can all benefit from good disease acceptance. Acceptance aims to increase a person's psychological flexibility and free them from the constraints of past occurrences. It can increase people's ability to act by their principles by replacing experienced avoidance with acceptance (Hayes et al. 2006; Hofmann, Friese, and Strack 2009). Clinical findings have shown that ACT intervention can successfully minimize empirical avoidance in the treatment of mental illnesses such as anxiety, depression, job burnout, weight management, pain, stigma, and so on (Hayes et al. 2006). ACT therapies also showed significant reductions in symptoms such as distress, emotional disturbances, physical pain, cognitive impairment, and traumatic responses, as well as significant increases in symptoms such as quality of life and psychological flexibility (Experiencing Breast Cancer: Bodily Identity in Illness and Gender Context n.d.; Fashler et al. 2018; Shari, Zainal, and Ng 2021).*

*4.3 ACT intervention and clinical symptoms*

*The ACT treatment method is quite flexible, and a suitable treatment plan can be based on the patient’s current situation (Wells and Fisher n.d.) to increase treatment acceptability. The purpose of ACT is not necessarily emotional and cognitive content management, but flexibility: encountering the present more fully as it is and continuing or modifying behavior in service of defined ideals. Furthermore, the ultimate purpose of ACT is not to relieve patients' clinical symptoms, but to improve their ability to choose acts consistent with their values by increasing psychological flexibility, which frequently leads to symptom relief. ACT is less concerned with the theory and more concerned with metaphorical storytelling and experiential activities, which improves the treatment's effectiveness. Rather than addressing a specific symptom, ACT focuses on healing the interaction between people and their unpleasant emotions, thought patterns, and behaviors. As a result, ACT's intervention is more effective when dealing with comorbid diseases (Wolitzky-Taylor et al. 2012).*

**Theoretical framework**

***Lazarus’s Transactional theory and research one motions and coping***

*The transactional theory proposed by Lazarus and Folkman （Lazams RS,1987） is a paradigm of stress and coping. According to this theory, stress results when a transaction between an individual and his environment is appraised by the individual as exceeding his resources and endangering his well-being. What relates to well-being is not the mere objective presence of stressful demands in the environment but rather the individual’s cognitive appraisal of these demands and his resources, as well as his repertoire of coping strategies. These are defined as cognitive or behavioral efforts to manage stressful demands.*


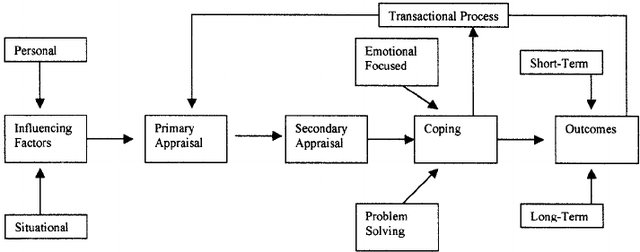


*Figure 1. Appraisal and coping model.*

**Conceptual framework**

*The research is based on* *Lazarus’s Transactional theory (Lazarus and Folkman 1987) of stress, and acceptance commitment therapy (Hayes et al. 2006) is the main intervention strategy. If a person is confronted with a breast cancer diagnosis, the preprogramed emotion is automatically generated as a feature of our biological heritage. ACT intervention as a kind of situational social support helps people improve their psychological flexibility, change their cognition of disease and improve their body image. Through ACT intervention to avoid anxiety and depression, and ultimately improve the quality of life. As a whole, they are interrelated and represent mental flexibility (Acceptance and Commitment Therapy, Second Edition: The Process and Practice ... - Steven C. Hayes, Kirk D. Strosahl, Kelly G. Wilson - Google book n.d.).*


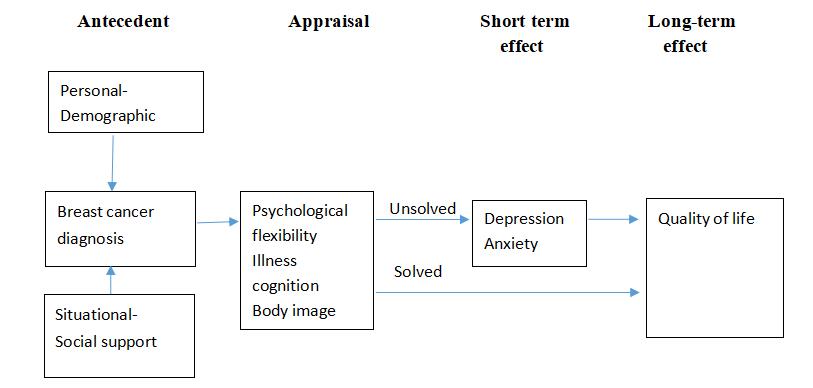


*Figure 2. Conceptual framework*

**Mediating and mediating model**

*The groups in the RCT study (ACT intervention and control) will be served as the independent variables. While the change scores of depression, anxiety, and quality of life from pre-treatment to post-assessment will be served as the dependent outcome. The change score is selected as a dependent variable instead of a post-assessment score to examine the improvement of the scores from pre to post-treatment. The scores of psychological inflexibility, illness cognition, and body image will be served as the proposed mediator variables.*

*In the moderating model, the groups in the RCT study (ACT intervention and control) will be served as the independent variables. The illness cognition, psychological flexibility, depression, anxiety, body image, and quality of life will be dependent variables and social support will be a moderator variable.*

*
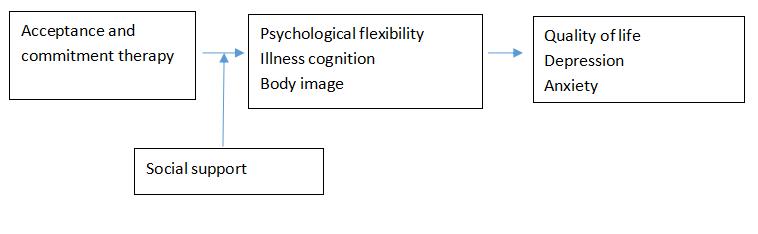
*

*Figure3.Mediation and moderation model*

**Research design**

**Study I:** Validation of BIS and ICQ

*This study examines the psychometric properties of the BIS and ICQ in the Malay version. The two questionnaires were obtained through the forward-backward translation method. This procedure aims to produce a translation, which can be easily read and understood, uses a common language, and remains conceptually equivalent to the original version.*

**Study II:** Efficacy of Acceptance and Commitment Therapy (ACT) on disease acceptance and quality of life in breast cancer patients

*This is a randomized controlled trial that examines the efficacy of brief acceptance and commitment therapy by comparing the differences between ACT intervention (cases) and waitlist (control).*

**Study area**

**Study I:**

*The respondents will be selected from the oncology clinics in Seberang Jaya hospital and Advanced Medical and Dental Institute (AMDI). The research will be carried out in a cancer clinic.*

**Study II:**

*The respondents will be selected from the oncology clinics in Seberang Jaya hospital and Advanced Medical and Dental Institute (AMDI). The study will take place in the wards and in the daily care centre’s consultation rooms.*

**Study population**

**Study I:**

*Cancer patients from the oncology clinic in Seberang Jaya hospital and AMDI.*

**Study II:**

*Breast cancer patients from Breast Clinic in Seberang Jaya hospital and AMDI.*

**Subject criteria:**

**Study I:**

*The inclusion criteria： Cancer patients must be 18 years of age or older, received a diagnosis of cancer, have basic writing, reading, and verbal communication abilities, and volunteer to participate in this study. Exclusion criteria included patients who were diagnosed with psychoses and substance abuse.*

**Study II:**

*The inclusion criteria are breast cancer patients must be 18 years old and above, newly diagnosed breast cancer patients in the treatment phase, have basic writing, reading, and verbal communication abilities, as well as a willingness to engage in the research, are required for admission. Patients with additional malignant tumors, patients who had already had ACT intervention, patients who had been given a diagnosis of psychosis, and substance abusers were among the exclusion criteria.*

**Sample size estimation**

**Study I:**

*The study intends to collect 400 questionnaires based on the sample size. This number is considered sufficient as the required sample size for a validation study is the number of items × 10 (Terwee CB, 2007).*

**Study II:**

*An a priori power analysis is computed using G*Power 3.1.9.222 to test the significant differences of measured variables between two independent groups whilst controlling the covariates. Based on the previous study, the sample size is determined based on the continuous response variable, Acceptance and action questionnaire (AAQ II), from a study conducted by Shari et al (Shari, Zainal, and Ng 2021) with a large effect size (0.61) and an alpha of 0.05, two-tailed. The result showed that a total sample of 75 respondents is required to achieve a power of 0.95. A total of 90 respondents for both groups with anticipation of a drop-out rate of 20%.*

**Sampling method and subject recruitment**

**Study I:**

*A purposive sampling method will be used to select subject matter experts to evaluate content validity.* *Find cancer patients in the IPPT outpatient clinic who are willing to complete the questionnaires. Before distributing the questionnaire, the researcher explained the study's purpose and significance to the patients. After obtaining informed consent, the researcher conducted the survey using the unified guidance language and required the patients to fill out the questionnaire independently.*

**Study II:**

*Patients with breast cancer should be referred by doctors at breast cancer clinics. The researcher briefed the patients about the relevance and goal of the study before distributing the questionnaire. The patients had to complete the survey on their own once the researcher had obtained their informed consent. The survey was carried out using uniform instruction language. Respondents will be randomly recruited based on odd and even sequences. Then, randomization of respondents into the intervention group (ACT) and the control group (treatment-as-usual controls) is carried out by a research assistant not involved in this study in a ratio of 1:1. To ensure that responders will receive a similar intervention, we will perform the study by the ACT module, which we currently have. The ACT module is adapted from a brief ACT module by Shari et al (Shari et al., 2021).*

**Research tool**

**Study I:**

*1. General Situation Questionnaire includes a demographic index: age, race, educational background, marital status, and employment status of the patients. Besides, the disease information will be obtained including sickness site, surgical method, disease stage, current treatment, and duration after diagnosis.*

*2. Malay version of Body Image Scale.*

*3. Malay version of Illness Cognition Scale***.**

**Study Ⅱ:**

*1. General Situation Questionnaire includes a demographic index: age, race, educational background, marital status, and employment status of the patients. Besides, the disease information will be obtained including sickness site, surgical method, disease stage, current treatment, and duration after diagnosis.*

*2. The Hospital Anxiety and Depression Scale (HADS), compiled by Zigmond and Snaith, is mainly used for screening non-psychotic anxiety and depression symptoms in hospitalized patients, including two subscales of anxiety and depression (Zigmond and Snaith 1983). HADS consist of 14 items: 7 items for anxiety and 7 items for depression.*

*3. The Acceptance and Action Questionnaire Ⅱ (AAQ-Ⅱ) Malay version measured psychological inflexibility or experiential avoidance. AAQ II consists of 7 items. A higher score on AAQ-Ⅱ indicates a greater level of psychological inflexibility (Shari et al. 2019).*

*4. The Malay version of the Illness Cognition Questionnaire (ICQ) evaluates disease cognition from both positive and negative perspectives, including three dimensions of helplessness, acceptance, and perception benefit. The higher the score, the stronger the condition of the corresponding dimensions (Evers et al. 2001).*

*5. The Functional Assessment of Cancer Therapy-Breast (FACT-B) consists of 5 dimensions including physical condition, social and family condition, emotional condition, functional condition, and breast cancer-specific module. The FACT-B consists of 37 items, the higher the score, the better the overall survival treatment and the corresponding dimension (Cella et al. 1993).*

*6. The Perceived Social Support Scale (PSSS) compiled by Blumenthal, includes two dimensions of support within the family and support outside the family, respectively measuring the various kinds of social support perceived by individuals. Meanwhile, the total score reflects the total degree of social support perceived by individuals. The PSSS consists of 18 items and the higher the score, the better the patient's perceived social support (Blumenthal et al. 1987).*

*7. Malay version of the Body image scale (BIS) is a brief questionnaire for assessing body image changes in patients with cancer, it consists of 10 items.*

**Interventions**

*Acceptance and commitment therapy (ACT)*

*Acceptance and commitment therapy (ACT) is one of the most representative experiential behavior therapies in the third wave of Cognitive and Behavior Therapy, named “contextual cognitive-behavioral therapy”. ACT aims to enhance psychological flexibility to help individuals devote themselves to a valuable and meaningful life, with mindfulness, acceptance, defusion, self as context, value, and committed action as well as flexible treatment techniques. Mohabbat-Bahar applied ACT group intervention to breast cancer patients, focusing on solving patients' anxiety and depression. In this study, 30 patients with breast cancer were randomly divided into intervention groups and control groups by a convenient sampling method. The interventions include evaluating cognitive fusion and values, accepting negative emotions and experiences, dissociating from personal experience, contact with the present and meditation training, determining values, making behavior plans, and so on. Patients in the control group received routine nursing. The study found that ACT group intervention can reduce anxiety and depression scores in patients with breast cancer (Moradi-Joo et al., n.d.).*

*Intervention content*

*Introductory session: The first step is that we should establish a good therapeutic relationship. Therapists can first introduce themselves to their professional background to establish a relationship of trust. Next, we will conduct an initial intake interview to encourage members to get to know each other. It contains the address, age, duration of illness, and changes in body appearance caused by loss of breasts caused by surgery, any disturbing thoughts and feeling related to her disease, predictors, precipitating factors, coping strategies, and its efficacy. And then we should develop a case formulation and map the respondent’s issues in life on a matric diagram. Understand and evaluate the patients' bad psychological status, and understand and master the patients' inner feelings and their ineffective strategies in the course of listening and observation. Finally, we will introduce our intervention project which is an overview of how ACT would be able to help to manage unpleasant thoughts and feelings from different perspective.*

*Let it go: Initially, according to defusion-creative hopelessness, let patients know that it is impossible to escape. Only accepting the facts can help us get out of a predicament. In the face of bad things and unpleasant thoughts, we should alternative to control is the willingness to give space and “sit” with the unpleasant thought, feeling, and memory. Learn to live with these bad things and unpleasant thoughts normally instead of getting rid of them. The correct state is actively “contact” with her psychological experiences without struggling. Metaphorical methods can be used to guide patients' feelings and establish defenseless attitudes. With the help of hypothetical sensory descriptions such as tug-of-war with a monster and physicalizing, it helps patients establish and improve their non-critical acceptance of reality and their own experience at the moment. To assess effects of physical changes and dysfunction after the operation and try to live with it. Besides, metaphors such as hands trap, the mind is a bully, passengers on the bus, and pushing paper, help people realize that they should defusion to reduce behavioral avoidance from unpleasant emotions and thoughts. Finally, form an objective perception of the disease, observe the experience external, face it bravely, do not fight and avoid it, and actively accept the effects of physical changes and dysfunction after the operation. At last, we give patients 2 home practices including an acceptance and defusion worksheet.*

*Show up: Talk about being in the present moment and self as context. In the language of daily life, when we talk about "thinking", we do not realize that it contains two parts: the thinking self and the observational self. We are all familiar with the thinking self, and this part of us is always thinking, such as generating thoughts, beliefs, memories, judgments, fantasies, plans, and so on. But most of us are not familiar with the observational self: no matter what we think, what we do, what we feel, or what we perceive, we can be aware of the observational self at any time. Another expression of the observational self is also called "total awareness". In the field of ACT, it is called "taking oneself as the scene" (self-as-context). For example, as you go through different stages of your life, your body can change, your thoughts can change, your feelings can change, and your role will change. But "you" can pay attention to and observe everything that has changed, and "you" has not changed at this point. This is a constant "you", life-long unchanged "you". In the face of visitors, we often use the term "observational self" to refer to "taking oneself as the scene". And then we will practice mindful breathing together and be encouraged to experience the thoughts and feeling with openness, interest, and receptiveness without attempting to change them. Guide patients to pay attention to and perceive their current situation and inner activities as a bystander and in a non-evaluation way, actively face and experience the present, and enjoy the care of medical staff, relatives and friends. Through metaphorical practice, like an emotional storm and dropping anchor, chessboard, and furnished room, the patient was asked to develop a position from which she was able to observe her unpleasant thoughts and feelings and let them come and go. Guide patients to treat their thoughts flexibly, treat them and emotions as words, rather than the facts themselves, calmly observe their own bad emotions, and reduce their fear of disease and worry about their future life in the process of cognitive dissociation. At last, we give patients 4 home practices including mindfulness exercise, mindful breathing, mindful eating, and mindful bathing.*

*Get moving: Determine values, goals and list strategies to achieve the goal and serve values. Through objective data, patients are informed of the development and progress of clinical diagnosis and treatment of breast cancer, the improvement of clinical survival rate, and the improvement of prognosis, to help patients adjust their mentality and further encourage patients to describe their self-yearning life. And then guide them to take the initiative to find, choose and make clear the direction of life in the future, help patients understand the value and significance of life, and re-establish confidence in life. Practice during treatment, such as values assessment, values compass, and bull eye. Encourage the patient to take at least minimal steps or actions that could move her in the direction of her valued life. At last, we give patients 4 home practices including Values assessment, values, action list, and mindfulness exercise.*

| *Session* | *Element in hexaflex* | *Duration* |
| --- | --- | --- |
| *Introductory* | *The therapeutic relationship, initial intake interview, and introduction to ACT* | *1 hour* |
| *Show up* | *Contact with the present moment and self as context* | *1 hour* |
| *Let it go* | *Acceptance and defusion* | *1 hour* |
| *Get moving* | *Values and committed action* | *1 hour* |

table 1: ACT intervention

*The brief acceptance and commitment therapy will be conducted individually.* *The ACT module is adapted from a brief ACT module by Shari et al (Shari et al., 2021). The intervention consisted of 3 modules comprising the essential components of ACT. Every module consists of two elements in hexaflex. The modules will be covered in 4 sessions: with 1 hour in each session. The sessions will be conducted for 4 consecutive weeks.*

**Data collection method**

**Study I:**

*The investigator explained the purpose and significance of the study to the patients before distributing the questionnaire, and after obtaining informed consent, the patients were required to fill out the questionnaire independently.*

**Study II:**

*Once the breast cancer patients agree to participate, informed consent will be obtained. The therapist is a clinical psychologist, ACT trained. The psychology therapist is required to administer the intervention when patients are in the hospital for treatment. The intervention consisted of 3 modules comprising the essential components of ACT. Every module consists of two elements in hexaflex. The modules will be covered in 4 sessions: with 1 hour in each session. The sessions will be conducted for 4 consecutive weeks. The batteries of assessment were carried out at 3-time points; pre-intervention, post-intervention, and 3 months after the intervention. Respondents in the intervention group will receive 4 sessions of acceptance and commitment therapy, and the control group will only receive ACT intervention once data collection is completed.*

**Study flowchart**

Translation the questionnaire

Study I: Psychometric properties of BIS and ICQ

Administer questionnaire

Statistical analysis

Reliability and validity the questionnaire of BIS and ICQ


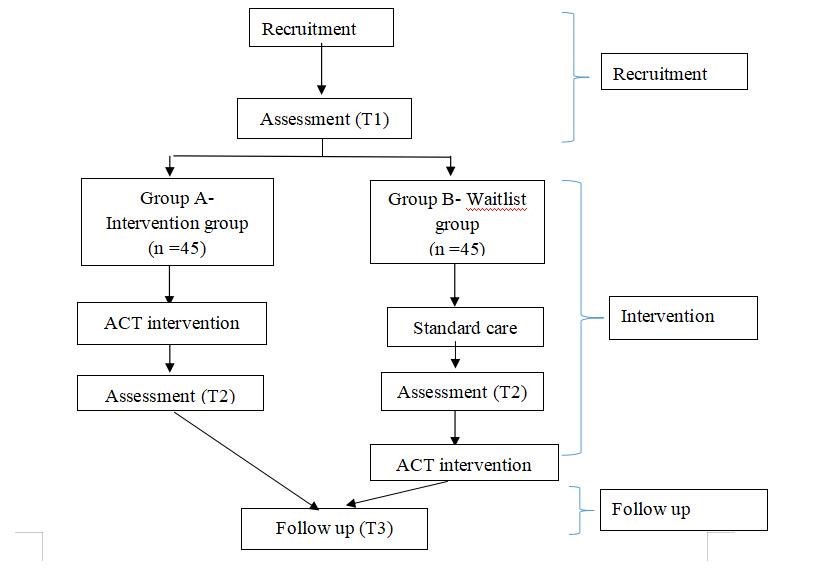


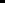


Study II: The effect of ACT on breast cancer patients

**Data analysis**

**Study I:**

*SPSS 22.0 will be used for statistical analysis. The general data of the subjects are described by (x s), frequency, and percentage. Cronbach's reliability coefficient and retest reliability test scale reliability were used, AMOS 21.0 will be used for confirmatory factor analysis, and structure validity and calibration validity were used. Examine the scale's reliability. 0.05 is considered significant.*

**Study II:**

*Data analyses will be carried out via a statistical package for social sciences (SPSS version 22). Descriptive statistics will be employed to analyse socio-demographic data. Frequency and percentage are used to describe race, age, marital status, educational background, employment status, and disease status including the stage at diagnosis, chemotherapy regime, and duration since diagnosis. Inference analysis will be used to examine the significant difference between waitlist and intervention groups before the implementation of ACT intervention.*

*The comparison of mean differences pre- and post-intervention will be examined to determine whether any changes in disease cognition, anxiety, depression, psychological flexibility, quality of life, body image, and social support among breast cancer patients in response to ACT intervention. Then, ANCOVA will be employed to examine the significant differences in measured variables between waitlist and intervention groups whilst controlling the covariates. The covariates will be identified based on the variables that have been significantly correlated with measured variables.*

*The effect sizes will be calculated to determine how substantially patients' perceptions towards measured variables changed with and without ACT intervention. The formula of Cohen’s d (Rosnow & Rosenthal, 1996) is used to calculate the effect size. Based on Cohen (1988), the effect size will consider as small if (d = 0.2), medium (d = 0.5), and large (d = 0.8).*

*Mediation analysis will be computed via PROCESS macro-Version 3.5 by Andrew F. Hayes. By using the single mediator model, the analysis will examine the total, direct, and indirect effects. The groups in the RCT study (ACT intervention and waitlist) will be the independent variables. While the change scores of illness cognition and quality of life from pre-treatment to post-assessment will be the dependent outcome. The change score is chosen as a dependent variable instead of a post-assessment score to examine the improvement of the scores from pre- to post-treatment. The scores of psychological inflexibilities, anxiety, depression, body image, and social support will be served as the proposed mediator variables. PROCESS will be used to verify the mediating effect of psychological flexibility and disease cognition between ACT intervention and quality of life, and the direct and indirect effects were verified by the Bootstrap program. Finally, social support is introduced into the coping path as a moderating variable to form a final model to verify the overall mediating effect.*

**Expected result(s)**

**Characteristics of patients**

| **Variables** | **Intervention(N=45)** | **Waitlist(N=45）** | **P value** |
| --- | --- | --- | --- |
| **Patient’s age (years) （Mean, SD)** |  |  |  |
| ≥ 18 |  |  |  |
| **Education Level, n(%)** |  |  |  |
| Primary |  |  |  |
| Secondary |  |  |  |
| Tertiary |  |  |  |
| **Employment Status, n(%)** |  |  |  |
| Employed |  |  |  |
| Unemployed |  |  |  |
| **Ethnic group, n(%)** |  |  |  |
| Malay |  |  |  |
| Chinese |  |  |  |
| Indian |  |  |  |
| Others |  |  |  |
| **Duration from diagnosis(month),n(%)** |  |  |  |
| 3 |  |  |  |
| 4 |  |  |  |
| 5 |  |  |  |
| 6 |  |  |  |
| **Stage, n(%)** |  |  |  |
| Stage 1 |  |  |  |
| Stage 2 |  |  |  |
| Stage 3 |  |  |  |

**Analyses of means differences between intervention and waitlist groups**

| **vatiables** |  | **Pre-treatment** | **Post-treatment** | **Means differences** | **Covariance analysis** | **Effect size** |
| --- | --- | --- | --- | --- | --- | --- |
| Psychological inflexibility | intervention |  |  |  |  |  |
|  | TAU |  |  |  |  |  |
| Anxiety | intervention |  |  |  |  |  |
|  | TAU |  |  |  |  |  |
| Depression | intervention |  |  |  |  |  |
|  | TAU |  |  |  |  |  |
| Body image | intervention |  |  |  |  |  |
|  | TAU |  |  |  |  |  |
| Social support | intervention |  |  |  |  |  |
|  | TAU |  |  |  |  |  |
| Quality of life | intervention |  |  |  |  |  |
|  | TAU |  |  |  |  |  |
| Disease acceptance | intervention |  |  |  |  |  |
|  | TAU |  |  |  |  |  |

TAU = treatment-as-usual control group

**Gantt chart & milestone**

| **Research Activities** | **2022** | | **2023** | | **2024** | |
| --- | --- | --- | --- | --- | --- | --- |
|  | **Jul –**  **Sep** | **Sep–**  **Dec** | **Jan- Jun** | **Jul-**  **Dec** | **Jan- Jun** | **Jul-**  **Dec** |
| Ethical application and approval from USM and NMRR |  |  |  |  |  |  |
| Data collection |  |  |  |  |  |  |
| Data analysis and interpretation |  |  |  |  |  |  |
| Thesis write-up and submission |  |  |  |  |  |  |

**Budget proposal [If applicable]:**

*This research proposal is still not funded yet.*

**Ethical consideration(s) [if applicable]:**

1. **Subject vulnerability**

*Since we also measure HADS so what if the respondents score moderate-to-severe depression and anxiety- will be referred to a psychiatrist. And the respondent is permitted to consult her family during the informed consent process.*

1. **Declaration of absence of conflict of interest**

*I declare that our study has no competing interests.*

1. **Privacy and confidentiality**

All forms are anonymous and will be entered into SPSS software. Only research team members can access the data. Data will be presented as grouped data and will not identify the responders individually.

1. **Community sensitivities and benefits**

*The completion of this psychological intervention study will be extremely beneficial to the rehabilitation and quality of life of breast cancer patients.*

1. **Honorarium and incentives**

*If this study received a grant, then an honorarium will be given to the respondents*

1. **Other ethical review board approval [if applicable]**

No

1. **Potential risk**

*To lessen injury, a psychological intervention will also be given to the control group.*

**References**

*“Acceptance and Commitment Therapy, Second Edition: The Process and Practice ... - Steven C. Hayes, Kirk D. Strosahl, Kelly G. Wilson - Google book.” https://books.google.com.hk/books?hl=zh-CN&lr=&id=og28CwAAQBAJ&oi=fnd&pg=PP1&dq=Acceptance+commitment+therapy+for+mindfulness+change&ots=-7NXXQ8Dsr&sig=ID-iXvFq5LgnAx1QoRcpQz8-tJw&redir_esc=y&hl=zh-CN&sourceid=cndr#v=onepage&q=Acceptance%20commitment%20therapy%20for%20mindfulness%20change&f=false (February 25, 2022).*

*Aerts, L. et al. 2014. “Sexual Functioning in Women after Mastectomy versus Breast Conserving Therapy for Early-Stage Breast Cancer: A Prospective Controlled Study.” Breast 23(5): 629–36.*

*Andersen, Barbara L. et al. 2008. “Psychologic Intervention Improves Survival for Breast Cancer Patients: A Randomized Clinical Trial.” Cancer 113(12): 3450–58.*

*Andrzej Nowicki, and Zaneta Ostrowska. 2008. “Disease Acceptance in Patients after Surgery from Breast Cancer during Supplementary Treatment.” Pol Merkur Lekarski 24(143): 403–7.*

*A-Tjak, Jacqueline G.L. et al. 2015. “A Meta-Analysis of the Efficacy of Acceptance and Commitment Therapy for Clinically Relevant Mental and Physical Health Problems.” Psychotherapy and Psychosomatics 84(1): 30–36. https://www.karger.com/Article/FullText/365764 (February 24, 2022).*

*A-Tjak, J G L et al. 2015. “A Meta-Analysis of the Efficacy of Acceptance and Commitment Therapy for Clinically Relevant Mental and Physical Health Problems.” Psychotherapy and Psychosomatics 84(1): 30–36. https://www.karger.com/DOI/10.1159/000365764.*

*Bakht, Sepideh, and Somayeh Najafi. 2010. “Body Image and Sexual Dysfunctions: Comparison between Breast Cancer Patients and Healthy Women.” In Procedia - Social and Behavioral Sciences, Elsevier Ltd, 1493–97.*

*Blumenthal, J. A. et al. 1987. “Social Support, Type A Behavior, and Coronary Artery Disease.” Psychosomatic medicine 49(4): 331–40. https://pubmed.ncbi.nlm.nih.gov/3615762/ (February 25, 2022).*

*Bower, Julienne E. et al. 2015. “Mindfulness Meditation for Younger Breast Cancer Survivors: A Randomized Controlled Trial.” Cancer 121(8): 1231–40. https://pubmed.ncbi.nlm.nih.gov/25537522/ (February 25, 2022).*

*“Cancer Research UK: Breast Cancer Survival Statistics.” https://www.cancerresearchuk.org/health-professional/cancer-statistics/statistics-by-cancer-type/breast-cancer (February 22, 2022).*

*Carroll, Brendan T et al. 1993. “Screening for Depression and Anxiety in Cancer Patients Using the Hospital Anxiety and Depression Scale.” General Hospital Psychiatry 15(2): 69–74. https://www.sciencedirect.com/science/article/pii/016383439390099A.*

*Carver, Charles S. 1997. “You Want to Measure Coping but Your Protocol’s Too Long: Consider the Brief COPE.” International journal of behavioral medicine 4(1): 92–100. https://pubmed.ncbi.nlm.nih.gov/16250744/ (February 25, 2022).*

*Cella, David F. et al. 1993. “The Functional Assessment of Cancer Therapy Scale: Development and Validation of the General Measure.” Journal of clinical oncology : official journal of the American Society of Clinical Oncology 11(3): 570–79. https://pubmed.ncbi.nlm.nih.gov/8445433/ (February 25, 2022).*

*Chang, Ya Lan et al. 2019. “Factors Influencing Body Image in Posttreatment Oral Cavity Cancer Patients.” Psycho-oncology 28(5): 1127–33. https://pubmed.ncbi.nlm.nih.gov/30889623/ (February 23, 2022).*

*Chen, S. C. et al. 2018. “Factors Associated with Healthcare Professional’s Rating of Disfigurement and Self-Perceived Body Image in Female Patients with Head and Neck Cancer.” European journal of cancer care 27(2). https://pubmed.ncbi.nlm.nih.gov/28488375/ (February 23, 2022).*

*Chen, Shuang Qin, Jun E. Liu, Zhi Li, and Ya Li Su. 2017. “The Process of Accepting Breast Cancer among Chinese Women: A Grounded Theory Study.” European journal of oncology nursing : the official journal of European Oncology Nursing Society 28: 77–85. https://pubmed.ncbi.nlm.nih.gov/28478860/ (February 25, 2022).*

*Chen, Wanqing et al. 2016. “Cancer Statistics in China, 2015.” CA: A Cancer Journal for Clinicians 66(2): 115–32. https://onlinelibrary.wiley.com/doi/full/10.3322/caac.21338 (February 28, 2022).*

*Cioffi, Delia, and James Holloway. 1993. “Delayed Costs of Suppressed Pain.” Journal of personality and social psychology 64(2): 274–82. https://pubmed.ncbi.nlm.nih.gov/8433273/ (February 25, 2022).*

*Civilotti, Cristina et al. 2021. “Anxiety and Depression in Women Newly Diagnosed with Breast Cancer and Waiting for Surgery: Prevalence and Associations with Socio-Demographic Variables.” Medicina (Lithuania) 57(5).*

*Cook, Sharon A. et al. 2018. “Predictors of Emotional Distress a Year or More after Diagnosis of Cancer: A Systematic Review of the Literature.” Psycho-Oncology 27(3): 791–801.*

*Cousson-Gélie, Florence, Marilou Bruchon-Schweitzer, Jean Marie Dilhuydy, and Marthe Aline Jutand. 2007. “Do Anxiety, Body Image, Social Support and Coping Strategies Predict Survival in Breast Cancer? A Ten-Year Follow-up Study.” Psychosomatics 48(3): 211–16. https://pubmed.ncbi.nlm.nih.gov/17478589/ (February 23, 2022).*

*Czerw, Aleksandra I., Magdalena Bilińska, and Andrzej Deptała. 2016. “The Assessment of the Impact of Socio-Economic Factors in Accepting Cancer Using the Acceptance of Illness Scale (AIS).” Wspolczesna Onkologia 20(3): 261–65.*

*Diego, Emilia J et al. 2016. “Axillary Staging After Neoadjuvant Chemotherapy for Breast Cancer: A Pilot Study Combining Sentinel Lymph Node Biopsy with Radioactive Seed Localization of Pre-Treatment Positive Axillary Lymph Nodes.” Annals of Surgical Oncology 23(5): 1549–53. https://doi.org/10.1245/s10434-015-5052-8.*

*Duijts, Saskia F A et al. 2011. “Effectiveness of Behavioral Techniques and Physical Exercise on Psychosocial Functioning and Health-Related Quality of Life in Breast Cancer Patients and Survivors—a Meta-Analysis.” Psycho-Oncology 20(2): 115–26. https://doi.org/10.1002/pon.1728.*

*Eifert, Georg H, and Michelle Heffner. 2003. “The Effects of Acceptance versus Control Contexts on Avoidance of Panic-Related Symptoms.” Journal of Behavior Therapy and Experimental Psychiatry 34(3): 293–312. https://www.sciencedirect.com/science/article/pii/S0005791603000570.*

*Elumelu, Theresa N., Chioma C. Asuzu, and Elizabeth O. Akin-Odanye. 2015. “Impact of Active Coping, Religion and Acceptance on Quality of Life of Patients with Breast Cancer in the Department of Radiotherapy, UCH, Ibadan.” BMJ supportive & palliative care 5(2): 175–80. https://pubmed.ncbi.nlm.nih.gov/25082529/ (February 25, 2022).*

*ESKELINEN, MATTI, and PAULA OLLONEN. 2011. “Assessment of ‘Cancer-Prone Personality’’ Characteristics in Healthy Study Subjects and in Patients with Breast Disease and Breast Cancer Using the Commitment Questionnaire: A Prospective Case–Control Study in Finland.’” Anticancer Research 31(11).*

*Evers, Andrea W.M. et al. 2001. “Beyond Unfavorable Thinking: The Illness Cognition Questionnaire for Chronic Diseases.” Journal of Consulting and Clinical Psychology 69(6): 1026–36. /record/2001-05666-016 (February 25, 2022).*

*“Experiencing Breast Cancer: Bodily Identity in Illness and Gender Context.” http://www.fnyjlc.com/EN/Y2014/V0/I2/85 (February 25, 2022).*

*Fashler, Samantha R., Aliza Z. Weinrib, Muhammad Abid Azam, and Joel Katz. 2018. “The Use of Acceptance and Commitment Therapy in Oncology Settings: A Narrative Review.” Psychological reports 121(2): 229–52. https://pubmed.ncbi.nlm.nih.gov/28836916/ (February 25, 2022).*

*Favez, Nicolas et al. 2016. “Distress and Body Image Disturbances in Women with Breast Cancer in the Immediate Postsurgical Period: The Influence of Attachment Insecurity.” Journal of health psychology 21(12): 2994–3003. https://pubmed.ncbi.nlm.nih.gov/26092841/ (February 23, 2022).*

*Fawzy, Fawzy I., Andrea L. Canada, and Nancy W. Fawzy. 2003. “Malignant Melanoma: Effects of a Brief, Structured Psychiatric Intervention on Survival and Recurrence at 10-Year Follow-Up.” Archives of General Psychiatry 60(1): 100–103. https://jamanetwork.com/journals/jamapsychiatry/fullarticle/207030 (February 24, 2022).*

*Fingeret, Michelle Cororve, Irene Teo, and Daniel E. Epner. 2014. “Managing Body Image Difficulties of Adult Cancer Patients: Lessons from Available Research.” Cancer 120(5): 633–41. https://pubmed.ncbi.nlm.nih.gov/24895287/ (February 23, 2022).*

*Flaxman, Paul Edward, J. T. (John T.) Blackledge, and Frank W. Bond. “Acceptance and Commitment Therapy : Distinctive Features.” : 172.*

*Gan, Chen et al. 2019. 9 Am J Cancer Res Neural Correlates of Chemotherapy-Induced Emotion Regulation Impairment in Breast Cancer Patients. www.ajcr.us/ISSN:2156-6976/ajcr0088923.*

*Gillanders, David T., Ashleigh K. Sinclair, Margaret MacLean, and Kirsten Jardine. 2015. “Illness Cognitions, Cognitive Fusion, Avoidance and Self-Compassion as Predictors of Distress and Quality of Life in a Heterogeneous Sample of Adults, after Cancer.” Journal of Contextual Behavioral Science 4(4): 300–311.*

*“Group Psychotherapy for Recently Diagnosed Breast Cancer Patients: A Multicenter Feasibility Study - Spiegel - 1999 - Psycho-Oncology - Wiley Online Library.” https://onlinelibrary.wiley.com/doi/10.1002/(SICI)1099-1611(199911/12)8:6%3C482::AID-PON402%3E3.0.CO;2-W (February 24, 2022).*

*Hack, Thomas F., and Lesley F. Degner. 2004. “Coping Responses Following Breast Cancer Diagnosis Predict Psychological Adjustment Three Years Later.” Psycho-oncology 13(4): 235–47. https://pubmed.ncbi.nlm.nih.gov/15054728/ (February 25, 2022).*

*Hadlandsmyth, Katherine et al. 2019. “A Single-Session Acceptance and Commitment Therapy Intervention among Women Undergoing Surgery for Breast Cancer: A Randomized Pilot Trial to Reduce Persistent Postsurgical Pain.” Psycho-Oncology 28(11): 2210–17.*

*Han, Jing et al. 2018. “Illness Cognitions and the Associated Socio-Demographic and Clinical Factors in Chinese Women with Breast Cancer.” European Journal of Oncology Nursing 32: 33–39.*

*Han, Jing, Jun E. Liu, Ya Li Su, and Hui Qiu. 2019. “Effect of a Group-Based Acceptance and Commitment Therapy (ACT) Intervention on Illness Cognition in Breast Cancer Patients.” Journal of Contextual Behavioral Science 14: 73–81.*

*Han, Jing, Li Zhang, Yao Zhang, and Ruijin Tang. 2021. “The Mediating Effect of Positive Illness Cognitions on Experiential Avoidance and Quality of Life in Breast Cancer Patients.” Asia-Pacific Journal of Oncology Nursing 8(4): 427–32.*

*Harris, Russ. 2009. “ACT Made Simple : An Easy-to-Read Primer on Acceptance and Commitment Therapy.” : 265.*

*Hayes, Steven C. et al. 1996. “Experiential Avoidance and Behavioral Disorders: A Functional Dimensional Approach to Diagnosis and Treatment.” Journal of Consulting and Clinical Psychology 64(6): 1152–68. /record/1996-07086-005 (February 24, 2022).*

*Hayes. 2006. “Acceptance and Commitment Therapy: Model, Processes and Outcomes.” Behaviour research and therapy 44(1): 1–25. https://pubmed.ncbi.nlm.nih.gov/16300724/ (February 25, 2022).*

*Hayes. 2016. “Acceptance and Commitment Therapy, Relational Frame Theory, and the Third Wave of Behavioral and Cognitive Therapies – Republished Article.” Behavior Therapy 47(6): 869–85.*

*Hernández, Diana Chavelas et al. 2015. “Impact of Rhinectomy on the Body Image in Nose Cancer Patients.” Gaceta Mexicana de Oncologia 14(1): 36–45.*

*Hofmann, Wilhelm, Malte Friese, and Fritz Strack. 2009. “Impulse and Self-Control From a Dual-Systems Perspective.” Perspectives on psychological science : a journal of the Association for Psychological Science 4(2): 162–76. https://pubmed.ncbi.nlm.nih.gov/26158943/ (February 25, 2022).*

*Hopwood, P., I. Fletcher, A. Lee, and S. al Ghazal. 2001. “A Body Image Scale for Use with Cancer Patients.” European journal of cancer (Oxford, England : 1990) 37(2): 189–97. https://pubmed.ncbi.nlm.nih.gov/11166145/ (February 25, 2022).*

*Horgan, Olga, Chris Holcombe, and Peter Salmon. 2011. “Experiencing Positive Change after a Diagnosis of Breast Cancer: A Grounded Theory Analysis.” Psycho-oncology 20(10): 1116–25. https://pubmed.ncbi.nlm.nih.gov/20734340/ (February 25, 2022).*

*Horwitz, Eric M. et al. 2001. “Impact of Surgery and Chemotherapy on the Quality of Life of Younger Women with Breast Carcinoma: A Prospective Study.” Cancer 92(5): 1288–98.*

*Hsu, Su Chin, Hsiu Hung Wang, Shu Yuan Chu, and Hsiu Fang Yen. 2010a. “Effectiveness of Informational and Emotional Consultation on the Psychological Impact on Women with Breast Cancer Who Underwent Modified Radical Mastectomy.” The journal of nursing research : JNR 18(3): 215–26.*

*Hsu, S. C., Wang, H. H., Chu, S. Y., & Yen, H. F. 2010b. “Effectiveness of Informational and Emotional Consultation on the Psychological Impact on Women with Breast Cancer Who Underwent Modified Radical Mastectomy.” The journal of nursing research : JNR 18(3): 215–26. https://pubmed.ncbi.nlm.nih.gov/20808081/ (February 25, 2022).*

*Lazams RS, Folkman S. Transactional theory and research one motions and coping[J]. Eur J Pers,1987,1(3):141-169．*

*Irvine, Diane et al. 1991. “Psychosocial Adjustment in Women with Breast Cancer.” Cancer 67(4): 1097–1117. https://pubmed.ncbi.nlm.nih.gov/1991258/ (February 23, 2022).*

*Janowski, Konrad, Małgorzata Tatala, Tomasz Jedynak, and Karolina Wałachowska. 2020. “Social Support and Psychosocial Functioning in Women after Mastectomy.” Palliative and Supportive Care 18(3): 314–21. https://www.cambridge.org/core/article/social-support-and-psychosocial-functioning-in-women-after-mastectomy/FA22865B2DA5419E7DF24B8F2CB05D26.*

*Jensen, Christian Gaden et al. 2014. “What to Listen for in the Consultation. Breast Cancer Patients’ Own Focus on Talking about Acceptance-Based Psychological Coping Predicts Decreased Psychological Distress and Depression.” Patient education and counseling 97(2): 165–72. https://pubmed.ncbi.nlm.nih.gov/25086446/ (February 25, 2022).*

*Jones, Salene M.W. et al. 2015. “Depression and Quality of Life before and after Breast Cancer Diagnosis in older Women from the Women’s Health Initiative.” Journal of cancer survivorship : research and practice 9(4): 620. /pmc/articles/PMC4547920/ (February 28, 2022).*

*Kissane, David W. et al. 2004. “Effect of Cognitive-Existential Group Therapy on Survival in Early-Stage Breast Cancer.” Journal of Clinical Oncology 22(21): 4255–60.*

*Kowalczyk, Robert et al. 2019. “Factors Affecting Sexual Function and Body Image of Early-Stage Breast Cancer Survivors in Poland: A Short-Term Observation.” Clinical breast cancer 19(1): e30–39. https://pubmed.ncbi.nlm.nih.gov/30385228/ (February 23, 2022).*

*Krok, Jessica L., Tamara A. Baker, and Susan C. McMillan. 2013. “Sexual Activity and Body Image: Examining Gender Variability and the Influence of Psychological Distress in Cancer Patients.” Journal of gender studies 22(4): 409. /pmc/articles/PMC3999973/ (February 28, 2022).*

*Lazarus, Richard S., and Susan Folkman. 1987. “Transactional Theory and Research on Emotions and Coping:” https://doi.org/10.1002/per.2410010304 1(3): 141–69. https://journals.sagepub.com/doi/pdf/10.1002/per.2410010304 (February 25, 2022).*

*Leak, Ashley, Jie Hu, and Cynthia R. King. 2008. “Symptom Distress, Spirituality, and Quality of Life in African American Breast Cancer Survivors.” Cancer nursing 31(1). https://pubmed.ncbi.nlm.nih.gov/18176122/ (February 23, 2022).*

*Lee, So Hyun, Yu Sun Min, Ho Yong Park, and Tae du Jung. 2012. “Health-Related Quality of Life in Breast Cancer Patients with Lymphedema Who Survived More than One Year after Surgery.” Journal of Breast Cancer 15(4): 449. /pmc/articles/PMC3542854/ (February 23, 2022).*

*Ludwigson, Abigail et al. 2020. “A Screening Tool Identifies High Distress in Newly Diagnosed Breast Cancer Patients.” Surgery 168(5): 935–41. http://www.surgjournal.com/article/S0039606020302634/fulltext (February 23, 2022).*

*Massie, Mary Jane. 2004. “Prevalence of Depression in Patients With Cancer.” JNCI Monographs 2004(32): 57–71. https://academic.oup.com/jncimono/article/2004/32/57/1021822 (February 23, 2022).*

*Mehnert, Anja et al. 2014. “Four-Week Prevalence of Mental Disorders in Patients With Cancer Across Major Tumor Entities.” Journal of Clinical Oncology 32(31): 3540–46. https://doi.org/10.1200/JCO.2014.56.0086.*

*Miller, Danny, and Isabelle le Breton-Miller. 2005. “Psychiatric Sequelae Following Breast Cancer Chemotherapy: A Pilot Study Using Claims Data.” Psychosomatics 46(6): 517–22.*

*Mitchell, Alex J, Stephen Kaar, Chris Coggan, and Joanne Herdman. 2008. “Acceptability of Common Screening Methods Used to Detect Distress and Related Mood Disorders—Preferences of Cancer Specialists and Non-Specialists.” Psycho-Oncology 17(3): 226–36. https://doi.org/10.1002/pon.1228.*

*Mokhatri-Hesari, Parisa, and Ali Montazeri. 2020. “Health-Related Quality of Life in Breast Cancer Patients: Review of Reviews from 2008 to 2018.” Health and Quality of Life Outcomes 18(1).*

*Moradi-Joo, Mohammad, Sahar Mohabbat-Bahar, Fatemeh Maleki-Rizi, and Mohammad Esmaeil Akbari. 8 Iran J Cancer Prev Effectiveness of Group Training Based on Acceptance and Commitment Therapy on Anxiety and Depression of Women with Breast Cancer.*

*Mosher, Catherine E. et al. 2020. “Protocol of a Randomized Trial of Acceptance and Commitment Therapy for Fatigue Interference in Metastatic Breast Cancer.” Contemporary Clinical Trials 98.*

*Park, Eliza M. et al. 2018. “Anxiety and Depression in Young Women With Metastatic Breast Cancer: A Cross-Sectional Study.” Psychosomatics 59(3): 251–58.*

*Pikler, Vanessa, and Carrie Winterowd. 2003. “Racial and Body Image Differences in Coping for Women Diagnosed with Breast Cancer.” Health psychology : official journal of the Division of Health Psychology, American Psychological Association 22(6): 632–37. https://pubmed.ncbi.nlm.nih.gov/14640861/ (February 23, 2022).*

*Riba, Michelle B. et al. 2019. “Distress Management, Version 3.2019.” JNCCN Journal of the National Comprehensive Cancer Network 17(10): 1229–49.*

*Roussi, Pagona, Vagia Krikeli, Christina Hatzidimitriou, and Ifigeneia Koutri. 2007. “Patterns of Coping, Flexibility in Coping and Psychological Distress in Women Diagnosed with Breast Cancer.” Cognitive Therapy and Research 2007 31:1 31(1): 97–109. https://link.springer.com/article/10.1007/s10608-006-9110-1 (February 25, 2022).*

*Shari, Nurul Izzah et al. 2019. “Psychometric Properties of the Acceptance and Action Questionnaire (AAQ II) Malay Version in Cancer Patients.” PloS one 14(2). https://pubmed.ncbi.nlm.nih.gov/30807594/ (February 25, 2022).*

*Shari, Nurul Izzah, Nor Zuraida Zainal, and Chong Guan Ng. 2021. “Effects of Brief Acceptance and Commitment Therapy (ACT) on Subjective Cognitive Impairment in Breast Cancer Patients Undergoing Chemotherapy.” Journal of psychosocial oncology 39(6): 695–714. https://pubmed.ncbi.nlm.nih.gov/33287685/ (February 25, 2022).*

*Silva, Araceli Vicente da, Eliana Zandonade, and Maria Helena Costa Amorim. 2017. “Anxiety and Coping in Women with Breast Cancer in Chemotherapy.” Revista Latino-Americana de Enfermagem 25.*

*Smith, Timothy B. et al. 2021. “Effects of Psychosocial Support Interventions on Survival in Inpatient and Outpatient Healthcare Settings: A Meta-Analysis of 106 Randomized Controlled Trials.” PLoS Medicine 18(5).*

*Speck, Rebecca M. et al. 2009. “Changes in the Body Image and Relationship Scale Following a One-Year Strength Training Trial for Breast Cancer Survivors with or at Risk for Lymphedema.” Breast Cancer Research and Treatment 2009 121:2 121(2): 421–30. https://link.springer.com/article/10.1007/s10549-009-0550-7 (February 23, 2022).*

*Stagl, Jamie M. et al. 2015. “Long-Term Psychological Benefits of Cognitive-Behavioral Stress Management for Women with Breast Cancer: 11-Year Follow-up of a Randomized Controlled Trial.” Cancer 121(11): 1873–81.*

*Stanton, Annette L., Sharon Danoff-burg, and Melissa E. Huggins. 2002. “The First Year after Breast Cancer Diagnosis: Hope and Coping Strategies as Predictors of Adjustment.” Psycho-oncology 11(2): 93–102. https://pubmed.ncbi.nlm.nih.gov/11921325/ (February 25, 2022).*

*Taleghani, Fariba, Zohreh Parsa Yekta, and Alireza Nikbakht Nasrabadi. 2006. “Coping with Breast Cancer in Newly Diagnosed Iranian Women.” Journal of Advanced Nursing 54(3): 265–72. https://onlinelibrary.wiley.com/doi/full/10.1111/j.1365-2648.2006.03808_1.x (February 24, 2022).*

*Tao, Wei Wei et al. 2015. “Psycho-Oncologic Interventions to Reduce Distress in Cancer Patients: A Meta-Analysis of Controlled Clinical Studies Published in People’s Republic of China.” Psycho-Oncology 24(3): 269–78.*

*Terwee CB, Bot SD, de Boer MR, van der Windt DA, Knol DL, Dekker J, Bouter LM, de Vet HC. Quality criteria were proposed for measurement properties of health status questionnaires. J Clin Epidemiol. 2007;60(1):34–42.*

*Tsaras, Konstantinos et al. 2018. “Assessment of Depression and Anxiety in Breast Cancer Patients: Prevalence and Associated Factors.” Asian Pacific Journal of Cancer Prevention 19(6): 1661–69.*

*Twohig, Michael P. 2012. “Acceptance and Commitment Therapy: Introduction.” Cognitive and Behavioral Practice 19(4): 499–507.*

*Vespa, Anna et al. 2012. “Evaluation of Intrapsychic Processes, Anxiety, and Depression in Postmenopausal Women Affected by Breast Cancer: A Case–Control Study.” Supportive Care in Cancer 2012 21:5 21(5): 1281–86. https://link.springer.com/article/10.1007/s00520-012-1659-5 (February 23, 2022).*

*Wang, Deming, Martin S. Hagger, and Nikos L.D. Chatzisarantis. 2020. “Ironic Effects of Thought Suppression: A Meta-Analysis.” Perspectives on psychological science : a journal of the Association for Psychological Science 15(3): 778–93. https://pubmed.ncbi.nlm.nih.gov/32286932/ (February 25, 2022).*

*Wells, Adrian., and Peter Fisher. “Treating Depression : MCT, CBT and Third Wave Therapies.”*

*Witek-Janusek, Linda et al. 2008. “Effect of Mindfulness Based Stress Reduction on Immune Function, Quality of Life and Coping in Women Newly Diagnosed with Early Stage Breast Cancer.” Brain, behavior, and immunity 22(6): 969–81. https://pubmed.ncbi.nlm.nih.gov/18359186/ (February 28, 2022).*

*Wolitzky-Taylor, Kate B., Joanna J. Arch, David Rosenfield, and Michelle G. Craske. 2012. “Moderators and Non-Specific Predictors of Treatment Outcome for Anxiety Disorders: A Comparison of Cognitive Behavioral Therapy to Acceptance and Commitment Therapy.” Journal of consulting and clinical psychology 80(5): 786–99. https://pubmed.ncbi.nlm.nih.gov/22823858/ (February 25, 2022).*

*Zhang, Hailing, Qinghua Zhao, Peiye Cao, and Guosheng Ren. 2017. “Resilience and Quality of Life: Exploring the Mediator Role of Social Support in Patients with Breast Cancer.” Medical Science Monitor 23: 5969–79.*

*Zigmond, A. S., and R. P. Snaith. 1983. “The Hospital Anxiety and Depression Scale.” Acta psychiatrica Scandinavica 67(6): 361–70. https://pubmed.ncbi.nlm.nih.gov/6880820/ (February 25, 2022).*

SOCIO-DEMOGRAPHIC AND CLINICAL DATA QUESTIONNAIRE

| Socio-demographic and Clinical Data Questionnaire | Date of attendance:  ---/---/---  Next follow up:  ---/---/--- | Research ID: |
| --- | --- | --- |
| 1. Gender(Jantina): | | |
| ◎Male(Lelaki) ◎Female(Perempuan) | | |
| 1. Age(Umur) | | |
| ◎18-25 years(tahun) ◎26-45years(tahun) ◎46-65 years(tahun)  ◎more than 65 years(tahun) | | |
| 1. Race(Bangsa): | | |
| ◎Malay(Melayu) ◎Chinese(Cina) ◎Indian(India) ◎Others(Lain-lain) | | |
| 1. Religion(Agama): | | |
| ◎Islam ◎Buddhism(Buddha) ◎Hindu ◎Christian(Kristian)  ◎Others(Lain-lain) | | |
| 1. Monthly income(Pendapatan bulanan): | | |
| ◎less than RM 4500(kurang daripada RM4500)  ◎RM4500-11000  ◎Above RM11000(lebih daripada RM11000) | | |
| 1. Marital Status(Status Perkahwinan): | | |
| ◎Married(Berkahwin)  ◎Unmarried/Divorce/Widow/Widower(Belum berkahwin/Janda/Duda) | | |
| 1. Education Status(Status pelajaran): | | |
| ◎Until Primary School(Sehingga sekolah rendah)  ◎Until Secondary School(Sehingga sekolah menengah)  ◎Until Tertiary Education(Sehingga pelajaran tertieri) | | |
| 1. Diagnosed(diagnos)   ◎breast cancer(kanker payudara)  ◎lung cancer(kanker paru-paru)  ◎head and neck cancer(kanker kepala)  ◎colon cancer(kanker kolon)  ◎others(Lain-Lain) | | |
| 1. Time since diagnosis(masa sehak diagnosa) | | |
| ◎3month(bulan) ◎6month(bulan)  ◎1year(tahun) ◎2year(tahun) ◎3year(tahun) | | |
| 1. Stage of cancer(Tahap kanser): | | |
| ◎Stage 1(Tahap 1) ◎Stage 2(Tahap 2) ◎Stage 3(Tahap 3) | | |
| 1. Current treatment | | |
| ◎chemo 1( kemo 1) ◎chemo 2( kemo 2) ◎chemo 3( kemo 3) ◎others(Lain-Lain) | | |

*
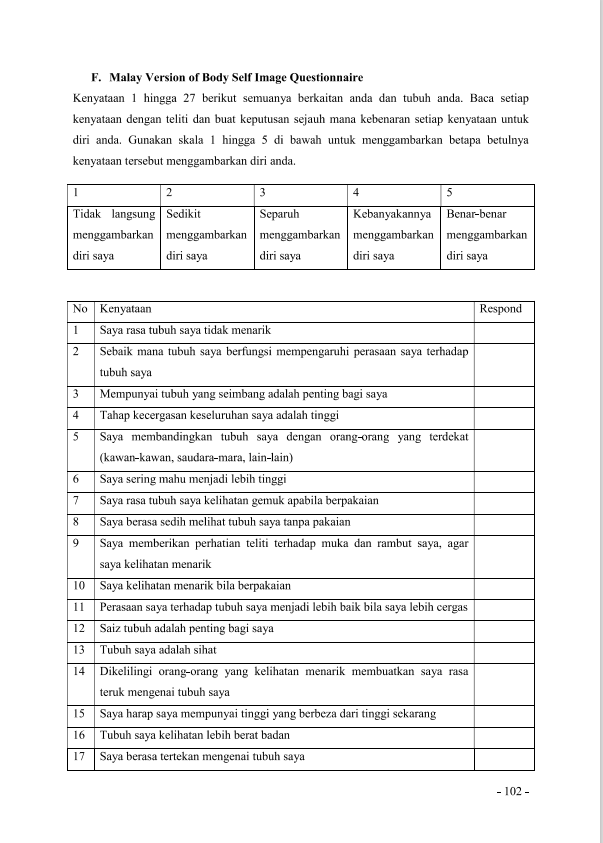
*

*
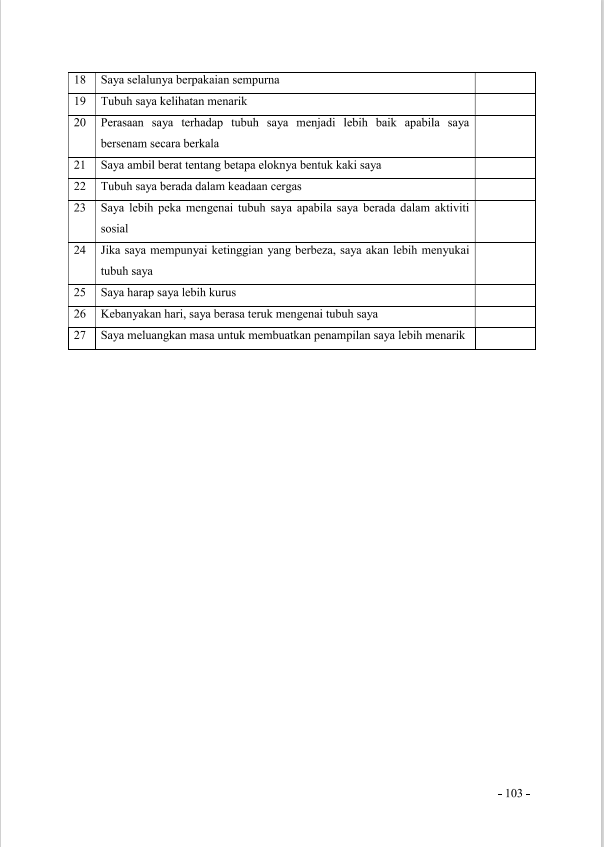
*

SOAL SELIDIK KOGNISI PENYAKIT

Arahan

Pada halaman seterusnya adalah senarai kenyataan daripada mereka yang menghidap penyakit kronik. Sila nyatakan tahap persetujuan anda dengan membulatkan salah satu jawapan berikutan pernyataan tersebut. Contoh adalah seperti di bawah.

Contoh

Jika anda bersetuju dengan kenyataan di bawah pada sebahagian besarnya, bulatan 3:

|  | Tidak setuju sama sekali | Agak setuju | Setuju pada sebahagian besarnya | Setuju sepenuhnya |
| --- | --- | --- | --- | --- |
| Saya telah belajar untuk hidup dengan penyakit saya. | 1 | 2 | 3 | 4 |

Jawab keseluruhan senarai pernyataan dengan cara ini. Jangan mengambil masa terlalu lama mempertimbangkan jawapan anda. Tanggapan pertama anda biasanya adalah yang terbaik.

**SOAL SELIDIK KOGNISI PENYAKIT**

| Sejauh manakah anda bersetuju dengan kenyataan berikut? | Tidak setuju sama sekali | Agak setuju | Setuju pada sebahagian besarnya | Setuju sepenuhnya |
| --- | --- | --- | --- | --- |
| 1. Kerana penyakit saya, saya amat merindui perkara yang saya suka lakukan. |  |  |  |  |
| 2. Saya boleh menangani masalah yang berkaitan dengan penyakit saya. |  |  |  |  |
| 3. Saya telah belajar untuk hidup dengan penyakit saya. |  |  |  |  |
| 4. Menangani penyakit menjadikan saya seorang yang lebih tabah. |  |  |  |  |
| 5. Penyakit saya mengawal hidup saya. |  |  |  |  |
| 6. Saya telah banyak belajar daripada penyakit saya. |  |  |  |  |
| 7. Kadangkala penyakit saya membuat saya berasa tidak berguna. |  |  |  |  |
| 8. Penyakit saya telah menjadikan hidup saya lebih berharga. |  |  |  |  |
| 9. Penyakit saya menghalang saya daripada melakukan apa yang saya sangat mahu lakukan. |  |  |  |  |
| 10. Saya telah belajar untuk menerima kesan penyakit yang membatasi hidup saya. |  |  |  |  |
| 11. Mengimbas kembali, saya dapat melihat bahawa penyakit saya juga membawa beberapa perubahan positif dalam hidup saya. |  |  |  |  |
| 12. Penyakit saya membatasi saya dalam segala hal yang penting bagi saya. |  |  |  |  |
| 13. Saya boleh menerima penyakit saya dengan baik. |  |  |  |  |
| 14. Saya rasa saya boleh menangani masalah yang berkaitan dengan penyakit saya walaupun penyakit itu semakin teruk. |  |  |  |  |
| 15. Penyakit saya kerap membuatkan saya berasa tidak berdaya. |  |  |  |  |
| 16. Penyakit saya telah membantu saya menyedari apa yang penting dalam hidup. |  |  |  |  |
| 17. Saya boleh mengatasi penyakit saya dengan berkesan. |  |  |  |  |
| 18. Penyakit saya telah mengajar saya untuk lebih menikmati saat ini. |  |  |  |  |

*
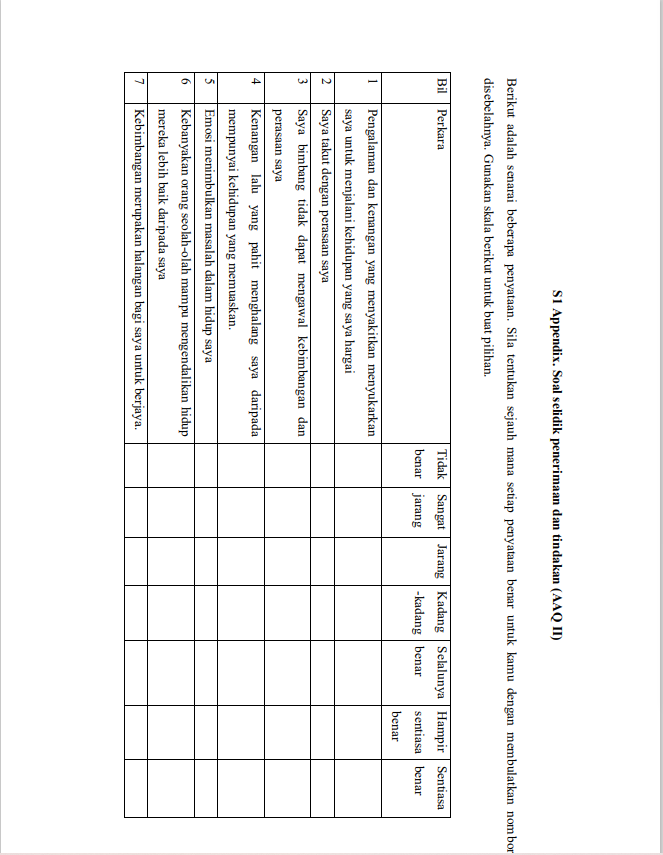
*

*
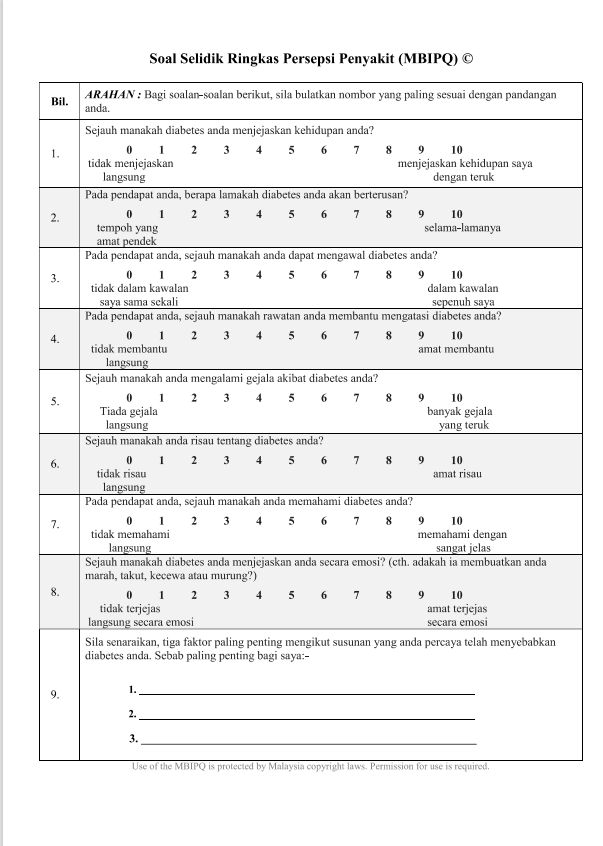
*

*
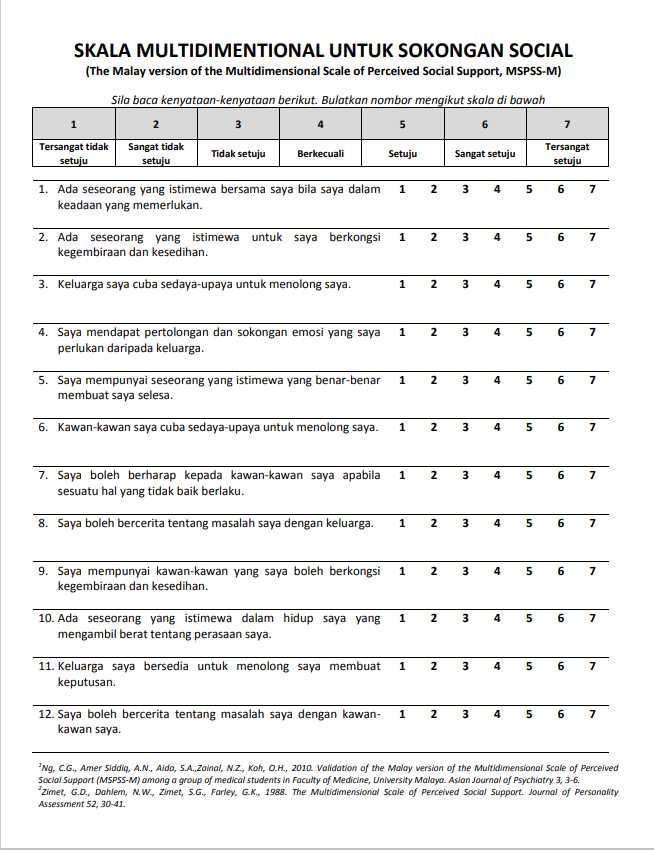
*

*
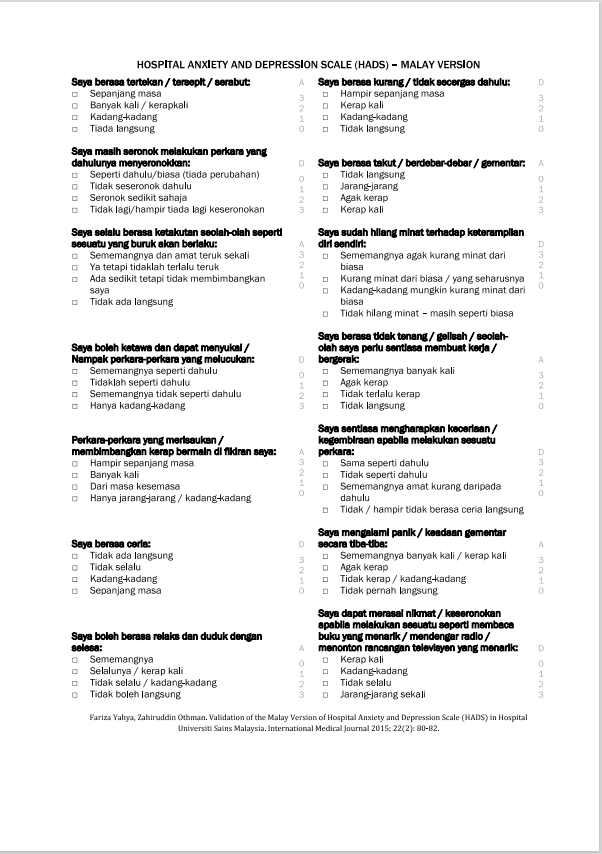
*

*
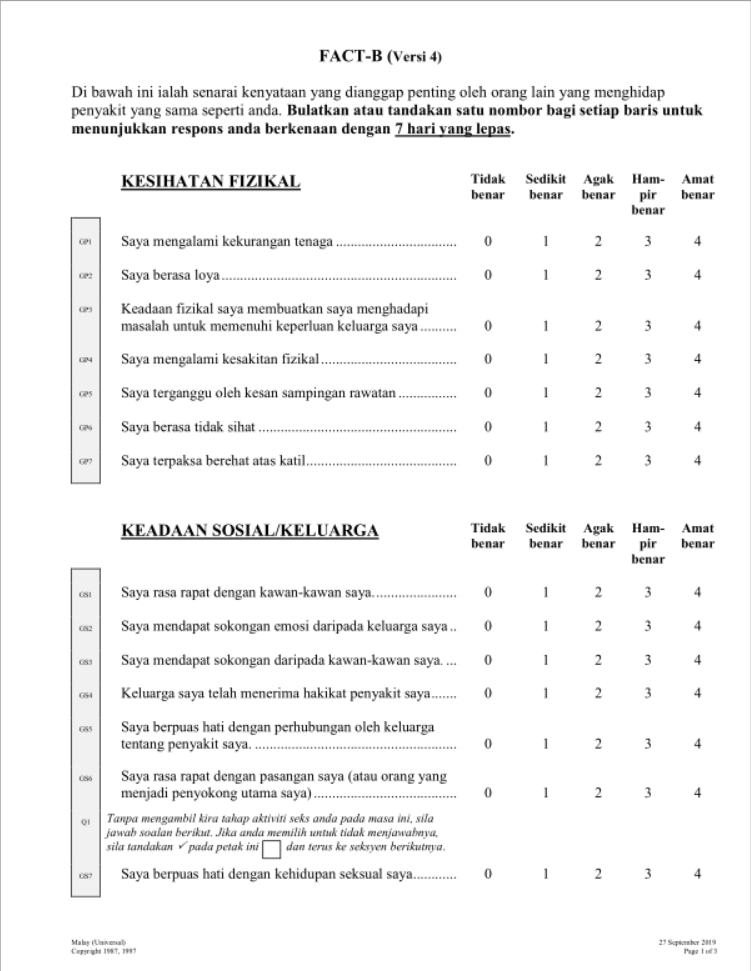
*

*
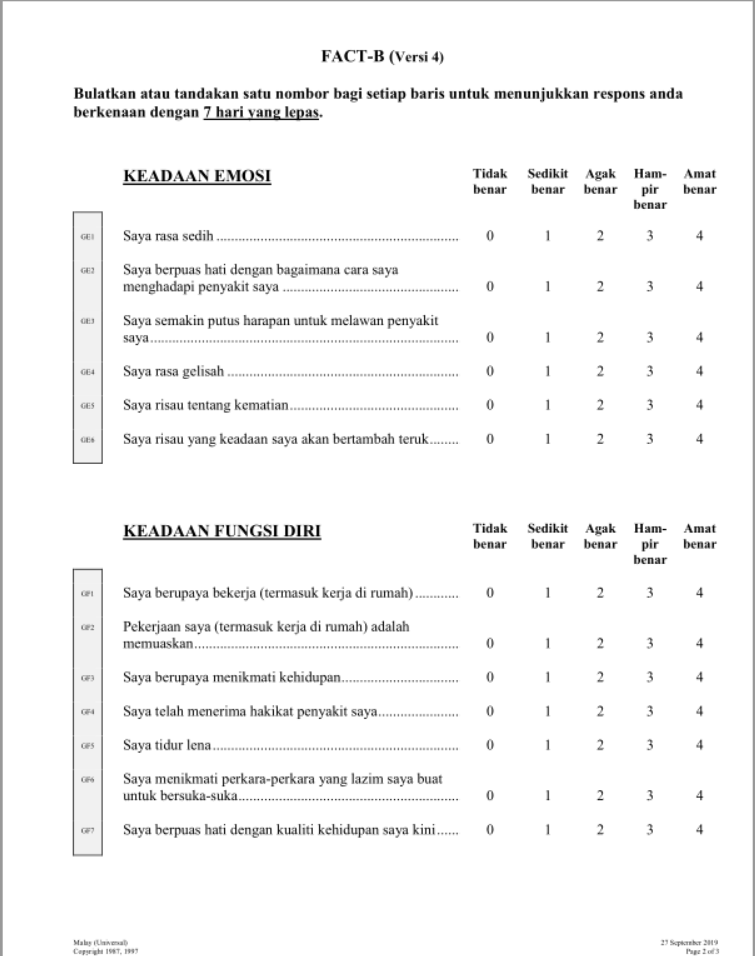
*

*
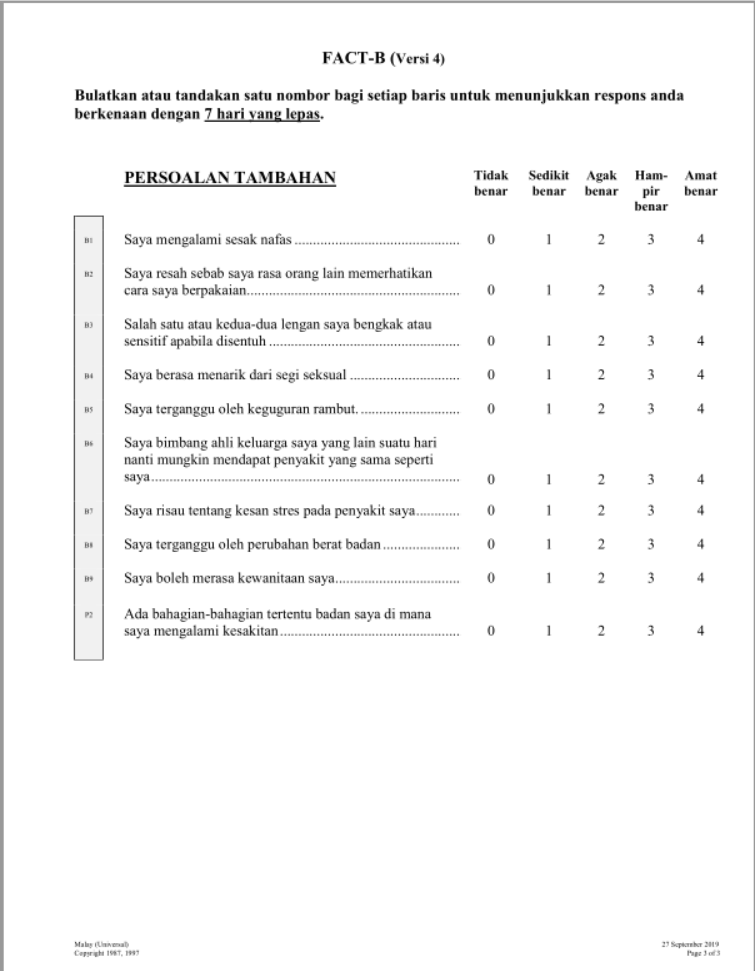
*


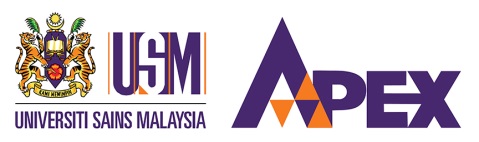


**JEPeM-USM**

JAWATANKUASA ETIKA PENYELIDIKAN (MANUSIA) – JEPeM USM

UNIVERSITI SAINS MALAYSIA

**LAMPIRAN A**

**MAKLUMAT KAJIAN I**

# Tajuk Kajian: Ciri-ciri psikometrik Soal Selidik Imej Badan versi Bahasa Melayu dan Soal Selidik Kognisi Penyakit pada Pesakit Kanser

# Nama Penyelidik dan penyelidik bersama: Song Wenjun, Nor Shuhada Murad @Mansor, Nurul Izzah Shari, Noor Mastura Mujar, Mohammad Farris Iman Leong Abdullah，Lu Ping

# PENGENALAN

Anda dijemput untuk mengambil bahagian secara sukarela dalam penyelidikan. Adalah penting anda membaca dan memahami maklumat penyelidikan ini sebelum bersetuju untuk menyertai kajian ini. Jika anda bersetuju untuk mengambil bahagian, anda akan menerima salinan borang ini untuk disimpan sebagai rekod anda.

Keadaan psikologi pesakit kanser biasanya dipengaruhi oleh diagnosis, komplikasi, dan kesan sampingan rawatan. Kajian ini ialah pengesahan soal selidik Imej Tubuh dan kognisi penyakit versi bahasa Melayu.

Untuk kajian ini, pengumpulan data mengambil masa 2 bulan dan sejumlah 400 peserta diperlukan

#### TUJUAN KAJIAN

**Kajian ini bertujuan untuk meneliti dan mengesahkan soal selidik imej tubuh dan kognisi penyakit versi bahasa Melayu.**

KELAYAKAN PENYERTAAN

#### Anda mesti benar-benar jujur dengan kakitangan penyelidik, termasuk sejarah kesihatan anda. Kriteria kemasukan: Pesakit kanser mestilah berumur 18 tahun ke atas, didiagnos dengan penyakit kanser, mempunyai kebolehan menulis, membaca dan komunikasi lisan asas, dan terlibat secara sukarela dalam kajian ini. Kriteria pengecualian termasuk pesakit yang didiagnosis dengan psikosis dan penyalahgunaan bahan.

#### PROSEDUR-PROSEDUR KAJIAN

**Penyelidik akan menjelaskan tujuan dan kepentingan kajian kepada pesakit sebelum mengedarkan borang soal selidik. Setelah mendapat persetujuan, pesakit dikehendaki mengisi soal selidik secara sendiri. Anggaran masa yang diperlukan untuk mengisi borang soal selidik adalah sekitar 15 hingga 20 minit.**

##### **RISIKO**

**Pesakit kanser bepotensi tinggi untuk menghadapi masalah kesihatan mental kerana diagnosis kanser dan rawatannya. Oleh itu, penyelidik akan memantau keadaan dan kerap menghubungi responden. Sila maklumkan kepada penyelidik jika anda menghadapi sebarang kemungkinan risiko dan masalah semasa menyertai kajian ini dan ingin menarik diri daripada kajian ini. Sekiranya keadaan menjadi lebih teruk, penyelidik akan merujuk responden untuk rawatan perubatan lanjut. Perbincangan dengan pakar psikiatri akan dibuat berkenaan perkara ini. Bagaimanapun, responden yang akan dirujuk kepada pakar psikiatri untuk rawatan perubatan akan dikeluarkan daripada kajian intervensi.**

PENYERTAAN DALAM KAJIAN

**Penglibatan anda dalam kajian ini adalah secara sukarela. Anda boleh menolak untuk mengambil bahagian dalam kajian atau anda boleh menghentikan penyertaan anda dalam kajian pada bila-bila masa, tanpa sebarang penalti atau kehilangan faedah yang anda berhak sebaliknya. Penyertaan anda juga boleh dihentikan oleh pasukan penyelidik tanpa kebenaran anda jika dalam apa jua bentuk anda telah melanggar kriteria kelayakan penyertaan. Ahli pasukan penyelidik akan berbincang dengan anda jika perkara itu timbul.**

MANFAAT YANG MUNGKIN [Manfaat terhadap Individu, Masyarakat, Universiti]

**Kajian ini akan diberikan kepada anda tanpa sebarang kos. Kajian ini memberi anda maklumat tentang cara mengurus emosi dan pemikiran sambil meningkatkan kesihatan mental. Kajian ini juga boleh membantu anda untuk menjadi lebih fleksibel dalam menghadapi cabaran dalam perjalanan kanser. Pesakit yang mempunyai kesihatan mental yang baik, daya tahan dan motivasi yang tinggi mempunyai kecenderungan yang lebih tinggi untuk berdaya tahan dalam trajektori kanser dengan baik.**

PERSOALAN

**Sekiranya anda mempunyai sebarang soalan mengenai prosedur kajian ini atau hak-hak anda, sila hubungi;**

Song Wenjun,

Community Health Department

Advanced Medical and Dental Institute,

Universiti Sains Malaysia

Tel.No.:0136794518

Email: [wenjun@student.usm.my](mailto:wenjun@student.usm.my)

ATAU

Nor Shuhada Murad

Community Health Department

Advanced Medical and Dental Institute,

Universiti Sains Malaysia

Tel.: +6013 586 6300

E-mail: shuhada.mansor@usm.my

**Sekiranya anda mempunyai sebarang soalan berkaitan kelulusan Etika atau sebarang pertanyaan dan masalah berkaitan kajian ini, sila hubungi;**

En. Mohd Bazlan Hafidz Mukrim

Setiausaha Jawatankuasa Etika Penyelidikan (Manusia) USM

Bahagian Penyelidikan dan Inovasi (P&I)

USM Kampus Kesihatan.

No. Tel: 09-767 2354 / 09-767 2362

Email : [bazlan@usm.my](mailto:bazlan@usm.my)

ATAU

Cik Nor Amira Khurshid Ahmed

Sekretariat Jawatankuasa Etika Penyelidikan (Manusia) USM

Pejabat Pengurusan dan Kreativiti Penyelidikan (RCMO)

USM Kampus Induk, Pulau Pinang.

No. Tel: 04-6536537

Email: noramira@usm.my

KERAHSIAAN

**Maklumat yang anda berikan akan dirahsiakan oleh kakitangan kajian. Ianya tidak akan dedahkan secara umum melainkan jika ia dikehendaki oleh undang-undang.**

**Data yang diperolehi dari kajian ini tidak akan mengenalpasti anda secara perseorangan. Hasil kajian mungkin akan diterbitkan untuk tujuan perkongsian ilmu.**

**Semua borang kajian dan data yang anda berikan yang asal mungkin akan disemak oleh pihak penyelidik, Lembaga Etika kajian ini dan pihak berkuasa regulatori bagi tujuan mengesahkan prosedur dan/atau data kajian klinikal. Maklumat anda akan disimpan dalam komputer dan hanya kakitangan kajian yang dibolehkan sahaja dibenarkan untuk mendapatkan dan memproses data tersebut.**

**Dengan menandatangani borang persetujuan ini, anda membenarkan penelitian rekod, penyimpanan maklumat dan pemprosesan data seperti yang dihuraikan di atas.**

TANDATANGAN

**Untuk dimasukkan ke dalam kajian ini, anda atau wakil sah anda mesti menandatangani serta mencatatkan tarikh halaman tandatangan (Lihat contoh Borang Keizinan Peserta di** LAMPIRAN S **dan** LAMPIRAN P**).**

LAMPIRAN S

Borang Keizinan Peserta

(Halaman Tandatangan)

***Tajuk Kajian:* Ciri-ciri psikometrik Soal Selidik Imej Badan versi Bahasa Melayu dan Soal Selidik Kognisi Penyakit pada Pesakit Kanser**

***Nama Penyelidik: Song Wenjun, Nor Shuhada Murad @Mansor, Nurul Izzah Shari, Noor Mastura Mujar, Mohammad Farris Iman Leong Abdullah, Lu Ping***

**Untuk menyertai kajian ini, anda atau wakil sah anda mesti menandatangani mukasurat ini. Dengan menandatangani mukasurat ini, saya mengesahkan yang berikut:**

- **Saya telah membaca semua maklumat dalam Borang Maklumat dan Keizinan Pesakit ini** termasuk apa-apa maklumat berkaitan risiko yang ada dalam kajian **dan saya telah pun diberi masa yang mencukupi untuk mempertimbangkan maklumat tersebut.**
- **Semua soalan-soalan saya telah dijawab dengan memuaskan.**
- **Saya, secara sukarela, bersetuju menyertai kajian penyelidikan ini, mematuhi segala prosedur kajian dan memberi maklumat yang diperlukan kepada doktor, para jururawat dan juga kakitangan lain yang berkaitan apabila diminta.**
- **Saya boleh menamatkan penyertaan saya dalam kajian ini pada bila-bila masa.**
- **Saya telah pun menerima satu salinan Borang Maklumat dan Keizinan Peserta untuk simpanan peribadi saya.**

Nama Peserta

No. Kad Pengenalan Peserta

**Tandatangan Peserta** atau Wakil Sah **Tarikh** (dd/MM/yy)

(Masa jika perlu)

Nama & Tandatangan Individu **yang Mengendalikan** Tarikh **(dd/MM/yy)**

**Perbincangan Keizinan**

Nama Saksi dan Tandatangan Tarikh **(dd/MM/yy)**

Nota: **i) Semua peserta yang mengambil bahagian dalam projek penyelidikan ini tidak dilindungi insuran.**

LAMPIRAN P

Borang Keizinan bagi Penerbitan Bahan yang berkaitan dengan Peserta Kajian

(Halaman Tandatangan)

*Tajuk Kajian:* Ciri-ciri psikometrik Soal Selidik Imej Badan versi Bahasa Melayu dan Soal Selidik Kognisi Penyakit pada Pesakit Kanser

*Nama Penyelidik: Song Wenjun, Nor Shuhada Murad @Mansor, Nurul Izzah Shari, Noor Mastura Mujar, Mohammad Farris Iman Leong Abdullah, Lu Ping*

**Untuk menyertai kajian ini, anda atau wakil sah anda mesti menandatangani mukasurat ini.**

**Dengan menandatangani mukasurat ini, saya memahami yang berikut:**

- **Bahan yang akan diterbitkan tanpa dilampirkan dengan nama saya dan setiap percubaan yang akan dibuat untuk memastikan ketanpanamaan saya. Saya memahami, walaubagaimanapun, ketanpanamaan yang sempurna tidak dapat dijamin. Kemungkinan sesiapa yang menjaga saya di hospital atau saudara dapat mengenali saya.**
- **Bahan yang akan diterbitkan dalam penerbitan mingguan/bulanan/dwibulanan/suku tahunan/dwi tahunan merupakan satu penyebaran yang luas dan tersebar ke seluruh dunia. Kebanyakan penerbitan ini akan tersebar kepada doktor-doktor dan juga bukan doktor termasuk ahli sains dan ahli jurnal.**
- **Bahan tersebut juga akan dilampirkan pada laman web jurnal di seluruh dunia. Sesetengah laman web ini bebas dikunjungi oleh semua orang.**
- **Bahan tersebut juga akan digunakan sebagai penerbitan tempatan dan disampaikan oleh ramai doktor dan ahli sains di seluruh dunia.**
- **Bahan tersebut juga akan digunakan sebagai penerbitan buku oleh penerbit jurnal.**
- **Bahan tersebut tidak akan digunakan untuk pengiklanan ataupun bahan untuk membungkus.**

**Saya juga memberi keizinan bahawa bahan tersebut boleh digunakan sebagai penerbitan lain yang diminta oleh penerbit dengan kriteria berikut:**

- **Bahan tersebut tidak akan digunakan untuk pengiklanan atau bahan untuk membungkus.**
- **Bahan tersebut tidak akan digunakan di luar konteks – contohnya: Gambar tidak akan digunakan untuk menggambarkan sesuatu artikel yang tidak berkaitan dengan subjek dalam foto tersebut.**

Nama Peserta

No. Kad Pengenalan Peserta **T/tangan Peserta** Tarikh **(dd/MM/yy)**

Nama & Tandatangan **Individu** **yang Mengendalikan**  Tarikh **(dd/MM/yy)**

**Perbincangan Keizinan**

Nota: **i) Semua peserta yang mengambil bahagian dalam projek penyelidikan ini tidak dilindungi insuran.**

CONTOH ATTACHMENT B

STUDY I INFORMATION

***Research Title:*** ***Psychometric properties of the Body Image Questionnaire and Illness Cognition Questionnaire Malay version in cancer patents***

***Name of main and co-Researchers: Song Wenjun, Nor Shuhada Murad @Mansor, Nurul Izzah Shari, Noor Mastura Mujar, Mohammad Farris Iman Leong Abdullah, Lu Ping***

#### INTRODUCTION

**You are invited to take part voluntarily in this research. It is important that you read and understand this research information before agreeing to participate in this study. If you agree to participate, you will receive a copy of this form to keep for your records.**

**The psychological state of cancer patients will be influenced by the diagnosis, complications, and treatment side effects. The study is the validation of the Body image and Malay Illness cognition questionnaire**

**For this study, data collection will take two months. This study is estimated to include up to 400 participants.**

#### PURPOSE OF THE STUDY

**This study aims to examine and validate the Body image and Malay Illness cognition questionnaire.**

#### PARTICIPANTS CRITERIA

**You must be completely truthful with the staff, including your health history. The inclusion criteria： Cancer patients must be 18 years of age or older, received a diagnosis of cancer, have basic writing, reading, and verbal communication abilities, and volunteer to participate in this study. Exclusion criteria included patients who were diagnosed with psychoses and substance abuse.**

STUDY PROCEDURES

**The investigator explained the purpose and significance of the study to the patients before distributing the questionnaire. Once informed consent is obtained, the patients are required to fill out the questionnaire independently estimated time required to fill in the questionnaire is around 15 to 20 minutes.**

RISKS

**Cancer patients are vulnerable to mental health issues due to cancer diagnosis and its treatments. Therefore, researchers will monitor the condition and regularly contact the respondents. Please inform the researcher if you encounter any possible risks and problems when participating in this study and want to withdraw from the study. If the condition becomes worst, researchers will refer the respondent for further medical treatment. A discussion with the psychiatrist will be made regarding this matter. However, respondents who will be referred to a psychiatrist for medical treatment will be removed from the intervention study.**

#### PARTICIPATION IN THE STUDY

**Your taking part in this study is entirely voluntary. You may refuse to take part in the study or you may stop your participation in the study at any time, without any penalty or loss of benefits to which you are otherwise entitled. Your participation also may be stopped by the research team without your consent if in any form you have violated the study eligibility criteria. The research team member will discuss this with you if the matter arises.**

#### POSSIBLE BENEFITS [Benefit to Individual, Community, University]

**This study will be provided to you at no cost. This study gives you information on how to manage emotions and thoughts while improving mental health. This study can also help you to become more flexible in facing the challenges in the breast cancer journey. Patients who have good mental health, resilience, and high motivation have a higher tendency to survive in the cancer trajectory well.**

#### QUESTIONS

**If you have any question about this study or your rights, please contact;**

Song Wenjun,

Community Health Department

Advanced Medical and Dental Institute,

Universiti Sains Malaysia

Tel.No.:0136794518

Email: [wenjun@student.usm.my](mailto:wenjun@student.usm.my)

OR

Nor Shuhada Murad

Community Health Department

Advanced Medical and Dental Institute,

Universiti Sains Malaysia

Tel.: +6013 586 6300

E-mail: shuhada.mansor@usm.my

**If you have any questions regarding the Ethical Approval or any issue / problem related to this study, please contact;**

Mr. Mohd Bazlan Hafidz Mukrim

Secretary of Human Research Ethics Committee USM

Division of Research & Innovation (R&I)

USM Health Campus

Tel. No. : 09-767 2354 / 09-767 2362

Email : [bazlan@usm.my](mailto:bazlan@usm.my)

OR

Miss Nor Amira Khurshid Ahmed

Secretariat of Human Research Ethics Committee USM

Research Creativity & Management Office (RCMO)

USM Main Campus, Penang

Tel. No. : 04-6536537

Email : [noramira@usm.my](mailto:noramira@usm.my)

#### CONFIDENTIALITY

**Your information will be kept confidential by the researchers and will not be made publicly available unless disclosure is required by law.**

**Data obtained from this study that does not identify you individually will be published for knowledge purposes.**

**Your original records may be reviewed by the researcher, the Ethical Review Board for this study, and regulatory authorities for the purpose of verifying the study procedures and/or data. Your information may be held and processed on a computer. Only research team members are authorized to access your information.**

**By signing this consent form, you authorize the record review, information storage and data process described above.**

#### SIGNATURES

**To be entered into the study, you or a legal representative must sign and data the signature page** [ATTACHMENT S and ATTACHMENT P]

ATTACHMENT S

Subject Information and Consent Form

(Signature Page)

*Research Title:* Psychometric properties of the Body Image Questionnaire and Illness Cognition Questionnaire Malay version in cancer patients

*Researcher’s Name:* Song Wenjun, Nor Shuhada Murad @Mansor, Nurul Izzah Shari, Noor Mastura Mujar, Mohammad Farris Iman Leong Abdullah, Lu Ping

**To become a part of this study, you or your legal representative must sign this page. By signing this page, I am confirming the following:**

- **I have read all of the information in this Patient Information and Consent Form** including any information regarding the risk in this study **and I have had time to think about it.**
- **All of my questions have been answered to my satisfaction.**
- **I voluntarily agree to be part of this research study, to follow the study procedures, and to provide necessary information to the doctor, nurses, or other staff members, as requested.**
- **I may freely choose to stop being a part of this study at any time.**
- **I have received a copy of this Participant Information and Consent Form to keep for myself.**

Participant Name

Participant I.C No

Signature of Participant **or Legal Representative** Date **(dd/MM/yy)**

Name of Individual

**Conducting Consent Discussion**

Signature of Individual Date **(dd/MM/yy)**

**Conducting Consent Discussion**

Name & Signature of Witness Date **(dd/MM/yy)**

**Note: i) All participants who are involved in this study will not be covered by insurance.**

ATTACHMENT P

Participant’s Material Publication Consent Form

Signature Page

*Research Title:* Psychometric properties of the Body Image Questionnaire and Illness Cognition Questionnaire Malay version in cancer patients

*Researcher’s Name: Song Wenjun, Nor Shuhada Murad @Mansor, Nurul Izzah Shari, Noor Mastura Mujar, Mohammad Farris Iman Leong Abdullah, Lu Ping*

**To become a part of this study, you or your legal representative must sign this page.**

**By signing this page, I am confirming the following:**

- **I understood that my name will not appear on the materials published and there have been efforts to make sure that the privacy of my name is kept confidential although confidentiality is not completely guaranteed due to unexpected circumstances.**
- **I have read the materials or general description of what the material contains and reviewed all photographs and figures in which I am included that could be published.**
- **I have been offered the opportunity to read the manuscript and to see all materials in which I am included but have waived my right to do so.**
- **All the published materials will be shared among medical practitioners, scientists, and journalists worldwide.**
- **The materials will also be used in local publications, and book publications and accessed by many local and international doctors worldwide.**
- **I hereby agree and allow the materials to be used in other publications required by other publishers with these conditions:**
- **The materials will not be used for advertising purposes nor as packaging materials.**
- **The materials will not be used out of context– i.e.: Sample pictures will not be used in an article that is unrelated subject to the picture.**

Participant Name

Participant I.C No. Participant’s Signature Date **(dd/MM/yy)**

Name and Signature of Individual Date **(dd/MM/yy)**

**Conducting Consent Discussion**

**Note: i) All participants who are involved in this study will not be covered by insurance.**


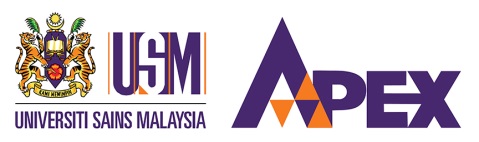


**JEPeM-USM**

JAWATANKUASA ETIKA PENYELIDIKAN (MANUSIA) – JEPeM USM

UNIVERSITI SAINS MALAYSIA

**LAMPIRAN A**

**MAKLUMAT KAJIAN II**

# Tajuk Kajian: Keberkesanan terapi penerimaan dan komitmen kepada penerimaan penyakit, kualiti hidup dikalangan pesakit kanser payudara: Percubaan terkawal rawak

# Nama Penyelidik dan penyelidik bersama: Song Wenjun, Nor Shuhada Murad @Mansor, Nurul Izzah Shari, Noor Mastura Mujar, Mohammad Farris Iman Leong Abdullah，Lu Ping

# PENGENALAN

Anda dijemput untuk mengambil bahagian secara sukarela dalam penyelidikan intervensi ini. Adalah penting anda membaca dan memahami maklumat penyelidikan ini sebelum bersetuju untuk menyertai kajian ini. Jika anda bersetuju untuk mengambil bahagian, anda akan menerima salinan borang ini untuk disimpan sebagai rekod anda.

Keadaan psikologi pesakit kanser biasanya dipengaruhi oleh diagnosis, komplikasi, dan kesan sampingan rawatan. Kajian terdahulu menunjukkan bahawa pesakit kanser payudara selepas mastektomi masih bergelut dengan penerimaan diagnosis mereka. Kajian juga menunjukkan bahawa psikoterapi secara berkesan boleh mengurangkan kesakitan psikologi pesakit kanser, melegakan kebimbangan dan kemurungan, dan meningkatkan kualiti hidup. Dengan perkembangan psiko-onkologi, intervensi psikologi semakin digunakan pada pesakit kanser payudara Kajian ini ialah kajian intervensi yang mengkaji keberkesanan terapi penerimaan dan komitmen terhadap penerimaan penyakit, kualiti hidup, fleksibiliti psikologi, kebimbangan, kemurungan, dan imej badan pada pesakit kanser payudara. Kajian ini juga mengkaji peranan sokongan sosial dalam membantu pesakit kanser payudara yang berhadapan dengan kanser.

Kajian ini akan selesai dalam tempoh 5 bulan dan akan mempunyai 90 peserta.

#### TUJUAN KAJIAN

**Kajian ini juga akan mengkaji keberkesanan terapi penerimaan dan komitmen terhadap penerimaan penyakit, kualiti hidup, fleksibiliti psikologi, kebimbangan, kemurungan, dan imej badan pada pesakit kanser payudara.**

*KELAYAKAN PENYERTAAN*

#### Anda mesti benar-benar jujur dengan kakitangan penyelidik, termasuk sejarah kesihatan anda. Kriteria inklusi adalah pesakit kanser payudara mestilah berumur 18 tahun ke atas, pesakit kanser payudara yang baru didiagnosis dalam fasa rawatan, asas menulis, membaca, dan komunikasi lisan kebolehan, serta kesediaan untuk melibatkan diri dalam penyelidikan diperlukan untuk kemasukan. Namun, pesakit dengan tumor malignan tambahan, pesakit yang telah pun menjalani campur tangan ACT, pesakit yang telah diberi diagnosis psikosis, dan penyalahgunaan bahan adalah antara kriteria pengecualian.

#### *PROSEDUR-PROSEDUR KAJIAN*

Sebaik sahaja pesakit kanser payudara bersetuju untuk mengambil bahagian dalam penyelidikan, persetujuan termaklum akan diambil. Ahli terapi adalah ahli psikologi klinikal, dan terlatih dalam ACT. Ahli terapi psikologi akan memberikan intervensi apabila pesakit berada di hospital untuk mendapatkan rawatan. Intervensi ini terdiri daripada 3 modul yang terdiri daripada komponen penting ACT. Modul akan diliputi dalam 4 sesi: dengan 1 jam untuk setiap sesi. Penilaian dan kutipan data akan dijalankan pada 3 masa; pra-intervensi, pasca intervensi, dan 3 bulan selepas intervensi. Dan kami akan mengumpulkan persetujuan termaklum sebanyak 2 kali, termasuk pra-intervensi, pasca-intervensi. Responden dalam kumpulan intervensi akan menerima 4 sesi terapi penerimaan dan komitmen, dan kumpulan kawalan hanya akan menerima intervensi ACT setelah pengumpulan data selesai**.**

##### RISIKO

Pesakit kanser payudara berpotensi tinggi untuk menghadapi masalah kesihatan mental kerana diagnosis kanser dan rawatannya. Oleh itu, penyelidik akan memantau keadaan dan kerap menghubungi responden. Sila maklumkan kepada penyelidik jika anda menghadapi sebarang kemungkinan risiko dan masalah semasa menyertai kajian ini dan ingin menarik diri daripada kajian ini. Sekiranya keadaan menjadi lebih teruk, penyelidik akan merujuk responden untuk rawatan perubatan lanjut. Perbincangan dengan pakar psikiatri akan dibuat berkenaan perkara ini. Bagaimanapun, responden yang akan dirujuk kepada pakar psikiatri untuk rawatan perubatan akan dikeluarkan daripada kajian intervensi.

PENYERTAAN DALAM KAJIAN

Penglibatan anda dalam kajian ini adalah secara sukarela. Anda boleh menolak untuk mengambil bahagian dalam kajian atau anda boleh menghentikan penyertaan anda dalam kajian pada bila-bila masa, tanpa sebarang penalti atau kehilangan faedah yang anda berhak sebaliknya. Penyertaan anda juga boleh dihentikan oleh pasukan penyelidik tanpa kebenaran anda jika dalam apa jua bentuk anda telah melanggar kriteria kelayakan penyertaan. Ahli pasukan penyelidik akan berbincang dengan anda jika perkara itu timbul.

MANFAAT YANG MUNGKIN [Manfaat terhadap Individu, Masyarakat, Universiti]

**Kajian ini akan diberikan kepada anda tanpa sebarang kos. Kajian ini memberi anda maklumat tentang cara mengurus emosi dan pemikiran sambil meningkatkan kesihatan mental. Kajian ini juga boleh membantu anda untuk menjadi lebih fleksibel dalam menghadapi cabaran dalam perjalanan kanser payudara. Pesakit yang mempunyai kesihatan mental yang baik, daya tahan dan motivasi yang tinggi mempunyai kecenderungan yang lebih tinggi untuk berdaya tahan dalam trajektori kanser dengan baik.**

PERSOALAN

**Sekiranya anda mempunyai sebarang soalan mengenai prosedur kajian ini atau hak-hak anda, sila hubungi;**

Song Wenjun,

Community Health Department

Advanced Medical and Dental Institute,

Universiti Sains Malaysia

Tel.No.:0136794518

Email: [wenjun@student.usm.my](mailto:wenjun@student.usm.my)

ATAU

Nor Shuhada Murad

Community Health Department

Advanced Medical and Dental Institute,

Universiti Sains Malaysia

Tel.: +6013 586 6300

E-mail: shuhada.mansor@usm.my

**Sekiranya anda mempunyai sebarang soalan berkaitan kelulusan Etika atau sebarang pertanyaan dan masalah berkaitan kajian ini, sila hubungi;**

En. Mohd Bazlan Hafidz Mukrim

Setiausaha Jawatankuasa Etika Penyelidikan (Manusia) USM

Bahagian Penyelidikan dan Inovasi (P&I)

USM Kampus Kesihatan.

No. Tel: 09-767 2354 / 09-767 2362

Email : [bazlan@usm.my](mailto:bazlan@usm.my)

ATAU

Cik Nor Amira Khurshid Ahmed

Sekretariat Jawatankuasa Etika Penyelidikan (Manusia) USM

Pejabat Pengurusan dan Kreativiti Penyelidikan (RCMO)

USM Kampus Induk, Pulau Pinang.

No. Tel: 04-6536537

Email: noramira@usm.my

KERAHSIAAN

**Maklumat yang anda berikan akan dirahsiakan oleh kakitangan kajian. Ianya tidak akan dedahkan secara umum melainkan jika ia dikehendaki oleh undang-undang.**

**Data yang diperolehi dari kajian ini tidak akan mengenalpasti anda secara perseorangan. Hasil kajian mungkin akan diterbitkan untuk tujuan perkongsian ilmu.**

**Semua borang kajian dan data yang anda berikan yang asal mungkin akan disemak oleh pihak penyelidik, Lembaga Etika kajian ini dan pihak berkuasa regulatori bagi tujuan mengesahkan prosedur dan/atau data kajian klinikal. Maklumat anda akan disimpan dalam komputer dan hanya kakitangan kajian yang dibolehkan sahaja dibenarkan untuk mendapatkan dan memproses data tersebut.**

**Dengan menandatangani borang persetujuan ini, anda membenarkan penelitian rekod, penyimpanan maklumat dan pemprosesan data seperti yang dihuraikan di atas.**

TANDATANGAN

**Untuk dimasukkan ke dalam kajian ini, anda atau wakil sah anda mesti menandatangani serta mencatatkan tarikh halaman tandatangan (Lihat contoh Borang Keizinan Peserta di** LAMPIRAN S **dan** LAMPIRAN P**).**

LAMPIRAN S

Borang Keizinan Peserta

(Halaman Tandatangan)

***Tajuk Kajian:* Keberkesanan terapi penerimaan dan komitmen kepada penerimaan**

**penyakit, kualiti hidup dikalangan pesakit kanser payudara: Percubaan**

**terkawal rawak**

***Nama Penyelidik:* Song Wenjun, Nor Shuhada Murad @Mansor, Nurul Izzah Shari, Noor Mastura Mujar, Mohammad Farris Iman Leong Abdullah，Lu Ping**

**Untuk menyertai kajian ini, anda atau wakil sah anda mesti menandatangani mukasurat ini. Dengan menandatangani mukasurat ini, saya mengesahkan yang berikut:**

- **Saya telah membaca semua maklumat dalam Borang Maklumat dan Keizinan Pesakit ini** termasuk apa-apa maklumat berkaitan risiko yang ada dalam kajian **dan saya telah pun diberi masa yang mencukupi untuk mempertimbangkan maklumat tersebut.**
- **Semua soalan-soalan saya telah dijawab dengan memuaskan.**
- **Saya, secara sukarela, bersetuju menyertai kajian penyelidikan ini, mematuhi segala prosedur kajian dan memberi maklumat yang diperlukan kepada doktor, para jururawat dan juga kakitangan lain yang berkaitan apabila diminta.**
- **Saya boleh menamatkan penyertaan saya dalam kajian ini pada bila-bila masa.**
- **Saya telah pun menerima satu salinan Borang Maklumat dan Keizinan Peserta untuk simpanan peribadi saya.**

Nama Peserta

No. Kad Pengenalan Peserta

**Tandatangan Peserta** atau Wakil Sah **Tarikh** (dd/MM/yy)

(Masa jika perlu)

Nama & Tandatangan Individu **yang Mengendalikan** Tarikh **(dd/MM/yy)**

**Perbincangan Keizinan**

Nama Saksi dan Tandatangan Tarikh **(dd/MM/yy)**

Nota: **i) Semua peserta yang mengambil bahagian dalam projek penyelidikan ini tidak dilindungi insuran.**

LAMPIRAN P

Borang Keizinan bagi Penerbitan Bahan yang berkaitan dengan Peserta Kajian

(Halaman Tandatangan)

*Tajuk Kajian:* Keberkesanan terapi penerimaan dan komitmen kepada penerimaan

penyakit, kualiti hidup dikalangan pesakit kanser payudara: Percubaan

terkawal rawak

*Nama Penyelidik:* Song Wenjun, Nor Shuhada Murad @Mansor, Nurul Izzah Shari, Noor Mastura Mujar, Mohammad Farris Iman Leong Abdullah，Lu Ping

**Untuk menyertai kajian ini, anda atau wakil sah anda mesti menandatangani mukasurat ini.**

**Dengan menandatangani mukasurat ini, saya memahami yang berikut:**

- **Bahan yang akan diterbitkan tanpa dilampirkan dengan nama saya dan setiap percubaan yang akan dibuat untuk memastikan ketanpanamaan saya. Saya memahami, walaubagaimanapun, ketanpanamaan yang sempurna tidak dapat dijamin. Kemungkinan sesiapa yang menjaga saya di hospital atau saudara dapat mengenali saya.**
- **Bahan yang akan diterbitkan dalam penerbitan mingguan/bulanan/dwibulanan/suku tahunan/dwi tahunan merupakan satu penyebaran yang luas dan tersebar ke seluruh dunia. Kebanyakan penerbitan ini akan tersebar kepada doktor-doktor dan juga bukan doktor termasuk ahli sains dan ahli jurnal.**
- **Bahan tersebut juga akan dilampirkan pada laman web jurnal di seluruh dunia. Sesetengah laman web ini bebas dikunjungi oleh semua orang.**
- **Bahan tersebut juga akan digunakan sebagai penerbitan tempatan dan disampaikan oleh ramai doktor dan ahli sains di seluruh dunia.**
- **Bahan tersebut juga akan digunakan sebagai penerbitan buku oleh penerbit jurnal.**
- **Bahan tersebut tidak akan digunakan untuk pengiklanan ataupun bahan untuk membungkus.**

**Saya juga memberi keizinan bahawa bahan tersebut boleh digunakan sebagai penerbitan lain yang diminta oleh penerbit dengan kriteria berikut:**

- **Bahan tersebut tidak akan digunakan untuk pengiklanan atau bahan untuk membungkus.**
- **Bahan tersebut tidak akan digunakan di luar konteks – contohnya: Gambar tidak akan digunakan untuk menggambarkan sesuatu artikel yang tidak berkaitan dengan subjek dalam foto tersebut.**

Nama Peserta

No. Kad Pengenalan Peserta **T/tangan Peserta** Tarikh **(dd/MM/yy)**

Nama & Tandatangan **Individu** **yang Mengendalikan**  Tarikh **(dd/MM/yy)**

**Perbincangan Keizinan**

Nota: **i) Semua peserta yang mengambil bahagian dalam projek penyelidikan ini tidak dilindungi insuran.**

ATTACHMENT B

RESEARCH INFORMATION II

***Research Title: The efficacy of Acceptance and Commitment Therapy (ACT) on disease acceptance and quality of life in breast cancer patients: A randomized controlled trial***

***Name of main and co-Researchers: Song Wenjun, Nor Shuhada Murad @Mansor, Nurul Izzah Shari, Noor Mastura Mujar, Mohammad Farris Iman Leong Abdullah，***Lu Ping

#### INTRODUCTION

You are invited to take part voluntarily in this interventional research. It is important that you read and understand this research information before agreeing to participate in this study. If you agree to participate, you will receive a copy of this form to keep for your records.

The psychological state of cancer patients will be influenced by the diagnosis, complications, and treatment side effects. Previous studies also demonstrated that breast cancer patients following mastectomy still struggle with acceptance of their diagnosis. Studies have shown that psychotherapy can effectively improve the psychological pain of cancer patients, relieve anxiety and depression, and improve quality of life. With the development of psycho-oncology, psychological intervention is increasingly used in patients with breast cancer. The study is an interventional study that examines the efficacy of acceptance and commitment therapy on disease acceptance, quality of life, psychological flexibility, anxiety, depression, and body image in patients with breast cancer. This study also examines the role of social support in helping breast cancer patients dealing with cancer.

The study will be finished within 8 months. This study is estimated to include up to 90 participants.

#### PURPOSE OF THE STUDY

This study will examine the efficacy of acceptance and commitment therapy on disease acceptance, quality of life, psychological flexibility, anxiety, depression, and body image in patients with breast cancer.

#### PARTICIPANTS CRITERIA

**You must be completely truthful with the staff, including your health history. The inclusion criteria are breast cancer patients must be 18 years old and above, newly diagnosed breast cancer patients in the treatment phase, basic writing, reading, and verbal communication abilities, as well as a willingness to engage in the research are required for admission. However, patients with additional malignant tumors, patients who had already had ACT intervention, patients who had been given a diagnosis of psychosis, and substance abusers were among the exclusion criteria.**

STUDY PROCEDURES

Once the breast cancer patients agree to participate, informed consent will be obtained. The therapist is a clinical psychologist, ACT trained. The psychology therapist will administer the intervention when patients are in the hospital for treatment. The intervention consisted of 3 modules comprising the essential components of ACT. The modules will be covered in 4 sessions: with 1 hour for each session. The batteries of assessment were carried out at 3-time points; pre-intervention, post-intervention, and 3 months after the intervention. And we will collect the informed consent 2 times, including pre-intervention, and post-intervention. Respondents in the intervention group will receive 4 sessions of acceptance and commitment therapy, and the control group will only receive ACT intervention once data collection is completed.

RISKS

Breast cancer patients are vulnerable to mental health issues due to cancer diagnosis and its treatments. Therefore, researchers will monitor the condition and regularly contact the respondents. Please inform the researcher if you encounter any possible risks and problems when participating in this study and want to withdraw from the study. If the condition becomes worst, researchers will refer the respondent for further medical treatment. Discussion with the psychiatrist will be made regarding this matter. However, respondents who will be referred to a psychiatrist for medical treatment will be removed from the intervention study.

#### PARTICIPATION IN THE STUDY

Your taking part in this study is entirely voluntary. You may refuse to take part in the study or you may stop your participation in the study at anytime, without any penalty or loss of benefits to which you are otherwise entitled. Your participation also may be stopped by the research team without your consent if in any form you have violated the study eligibility criteria. The research team member will discussed with you if the matter arises.

#### POSSIBLE BENEFITS [Benefit to Individual, Community, University]

This study will be provided to you at no cost. This study gives you information on how to manage emotions and thoughts while improving mental health. This study can also help you to become more flexible in facing the challenges in the breast cancer journey. Patients who have good mental health, resilience and high motivation have a higher tendency to survive in the cancer trajectory well.

#### QUESTIONS

**If you have any question about this study or your rights, please contact;**

Song Wenjun,

Community Health Department

Advanced Medical and Dental Institute,

Universiti Sains Malaysia

Tel.No.:0136794518

Email: [wenjun@student.usm.my](mailto:wenjun@student.usm.my)

OR

Nor Shuhada Murad

Community Health Department

Advanced Medical and Dental Institute,

Universiti Sains Malaysia

Tel.: +6013 586 6300

E-mail: shuhada.mansor@usm.my

**If you have any questions regarding the Ethical Approval or any issue / problem related to this study, please contact;**

Mr. Mohd Bazlan Hafidz Mukrim

Secretary of Human Research Ethics Committee USM

Division of Research & Innovation (R&I)

USM Health Campus

Tel. No. : 09-767 2354 / 09-767 2362

Email : [bazlan@usm.my](mailto:bazlan@usm.my)

OR

Miss Nor Amira Khurshid Ahmed

Secretariat of Human Research Ethics Committee USM

Research Creativity & Management Office (RCMO)

USM Main Campus, Penang

Tel. No. : 04-6536537

Email : [noramira@usm.my](mailto:noramira@usm.my)

#### CONFIDENTIALITY

**Your information will be kept confidential by the researchers and will not be made publicly available unless disclosure is required by law.**

**Data obtained from this study that does not identify you individually will be published for knowledge purposes.**

**Your original records may be reviewed by the researcher, the Ethical Review Board for this study, and regulatory authorities to verify the study procedures and/or data. Your information may be held and processed on a computer. Only research team members are authorized to access your information.**

**By signing this consent form, you authorize the record review, information storage, and data process described above.**

#### SIGNATURES

**To be entered into the study, you or a legal representative must sign and date the signature page** [ATTACHMENT S and ATTACHMENT P]

ATTACHMENT S

Subject Information and Consent Form

(Signature Page)

*Research Title:* The efficacy of Acceptance and Commitment Therapy (ACT) on disease acceptance and quality of life in breast cancer patients: A randomized controlled trial

*Researcher’s Name:* Song Wenjun, Nor Shuhada Murad @Mansor, Nurul Izzah Shari, Noor Mastura Mujar, Mohammad Farris Iman Leong Abdullah， L u Ping

**To become a part of this study, you or your legal representative must sign this page. By signing this page, I am confirming the following:**

- **I have read all of the information in this Patient Information and Consent Form** including any information regarding the risk in this study **and I have had time to think about it.**
- **All of my questions have been answered to my satisfaction.**
- **I voluntarily agree to be part of this research study, to follow the study procedures, and to provide necessary information to the doctor, nurses, or other staff members, as requested.**
- **I may freely choose to stop being a part of this study at any time**
- **I have received a copy of this Participant Information and Consent Form to keep for myself.**

Participant Name

Participant I.C No

Signature of Participant **or Legal Representative** Date **(dd/MM/yy)**

Name of Individual

**Conducting Consent Discussion**

Signature of Individual Date **(dd/MM/yy)**

**Conducting Consent Discussion**

Name & Signature of Witness Date **(dd/MM/yy)**

**Note: i) All participants who are involved in this study will not be covered by insurance.**

ATTACHMENT P

Participant’s Material Publication Consent Form

Signature Page

*Research Title:* The efficacy of Acceptance and Commitment Therapy (ACT) on disease acceptance and quality of life in breast cancer patients: A randomized controlled trial

*Researcher’s Name:* Song Wenjun, Nor Shuhada Murad @Mansor, Nurul Izzah Shari, Noor Mastura Mujar, Mohammad Farris Iman Leong Abdullah，Lu Ping

**To become a part of this study, you or your legal representative must sign this page.**

**By signing this page, I am confirming the following:**

- **I understood that my name will not appear on the materials published and there have been efforts to make sure that the privacy of my name is kept confidential although confidentiality is not completely guaranteed due to unexpected circumstances.**
- **I have read the materials or general description of what the material contains and reviewed all photographs and figures in which I am included that could be published.**
- **I have been offered the opportunity to read the manuscript and to see all materials in which I am included but have waived my right to do so.**
- **All the published materials will be shared among medical practitioners, scientists, and journalists worldwide.**
- **The materials will also be used in local publications, and book publications and accessed by many local and international doctors worldwide.**
- **I hereby agree and allow the materials to be used in other publications required by other publishers with these conditions:**
- **The materials will not be used for advertising purposes nor as packaging materials.**
- **The materials will not be used out of context – i.e.: Sample pictures will not be used in an article that is unrelated subject to the picture.**

Participant Name

Participant I.C No. Participant’s Signature Date **(dd/MM/yy)**

Name and Signature of Individual Date **(dd/MM/yy)**

**Conducting Consent Discussion**

**Note: i) All participants who are involved in this study will not be covered by insurance.**
